# Thrombin receptor PAR4 cross-activates the tyrosine kinase c-met in atrial cardiomyocytes

**DOI:** 10.1007/s00210-024-03436-6

**Published:** 2024-09-16

**Authors:** Claudia Mittendorff, Issam Abu-Taha, Lena Kassler, Tobias Hustedt, Stephanie Wolf, Johannes G. Bode, Markus Kamler, Dobromir Dobrev, Anke C. Fender

**Affiliations:** 1 Institute of Pharmacology, West German Heart and Vascular Center, University Duisburg-Essen, Duisburg, Germany; 2 Department of Gastroenterology, Hepatology and Infectious disease, Faculty of Medicine & Düsseldorf University Hospital, Heinrich-Heine-University, Düsseldorf, Germany; 3 Department of Thoracic and Cardiovascular Surgery, University Hospital Essen, Essen, Germany; 4 Department of Medicine and Research Center, Montreal Heart Institute and Université de Montréal, Montréal, Canada; 5 Department of Integrative Physiology, Baylor College of Medicine, Houston, TX USA

**Keywords:** Atrial, Cardiomyocyte, C-met, NLRP3 inflammasome, Protease-activated receptor, Thrombin

## Abstract

Thrombin supports coagulation-independent inflammation via protease-activated receptors (PAR). PAR4 is specifically increased in obese human atria, correlating with NLRP3 inflammasome activation. PAR4-mediated NLRP3 inflammasome activation in atrial cardiomyocytes is not known, nor have signaling partners been identified. Thrombin transactivates the hepatocyte growth factor receptor in some cancer cells, so we examined PAR4/c-met cross-talk in atrial cardiomyocytes and its possible significance in obesity. Cardiomyocytes from right atrial appendages (RAA) of obese patients expressed more PAR1 and PAR4 compared to non-obese. In HL-1 atrial cardiomyocytes, thrombin induced caspase-1 auto-activation and IL-1β maturation; IL-1β secretion was evoked by PAR4-activating peptide (AP), but not PAR1-AP. PAR4-AP additionally increased phosphorylated CaMKII-Thr287, mTOR-Ser2481, and Akt-Ser473 while suppressing AMPK-Thr172 phosphorylation. Total kinase levels were largely unaltered. PAR4AP rapidly increased phosphorylated c-met in HL-1 cells and over time also transcriptionally upregulated c-met. The c-met inhibitor SGX-523 abrogated the effects of PAR4-AP on CaMKII/AKT/mTOR phosphorylation but did not affect PAR4-stimulated IL-1β production. Obese human RAA contained more IL-1β, phospho-c-met, and phospho-mTOR than non-obese RAA; CamKII phosphorylation was not modified. Atria from high-fat diet (HFD) versus chow-fed mice also contained more IL-1β, together with higher myeloperoxidase activity, *Acta2* mRNA total and phosphorylated c-met; these increases were blunted in PAR4^-/-^ HFD-fed mice. Thrombin cross-activates c-met via PAR4 in atrial cardiomyocytes. Transactivated c-met contributes partially to PAR4-mediated signaling, but NLRP3 inflammasome activation appears to be largely independent of c-met. Abundance of PAR4 and activated c-met increases with obesity, providing therapeutic targets for management of adiposity-driven AF.

**Supplementary Information:**

The online version contains supplementary material available at 10.1007/s00210-024-03436-6.

## Introduction

The coagulant protease thrombin is a pivotal regulator of both hemostasis and inflammation and as such essentially links immunothrombosis with thromboinflammation (Stark and Massberg 2021, Schrottmaier and Assinger 2024). Yet, thrombin also supports sterile inflammatory signaling, independently of its pro-coagulant action, via cellular protease-activated receptors (PAR) (Fender et al. 2017, Fender et al. 2019). PAR1 is the prototypical thrombin receptor that responds to low levels of thrombin with rapid on-off kinetics (Sidhu et al. 2014), while PAR2 responds only to supra-physiological thrombin concentrations (Mihara et al. 2016), and PAR3 is thought to act primarily as a co-factor for other PAR. PAR4 is a low-affinity thrombin receptor that is dynamically regulated in response to oxidative and metabolic stress (Fender et al. 2017) and contributes to the pro-inflammatory actions of thrombin in the diabetic vasculature (Pavic et al. 2014), in obese adipose tissue (Kleeschulte et al. 2024), and in neurons after ischemic stroke (Fleischer et al. 2024). We could recently identify PAR4 as an upstream regulator of the cardiac NLRP3 inflammasome (Fender et al. 2020), a signaling platform for the auto-activation of caspase-1 and the subsequent maturation and secretion of interleukin (IL)-1β. In atrial myocardium, over-abundance and activity of the NLRP3 inflammasome complex specifically within cardiomyocytes is causally linked with atrial fibrillation (AF) (Yao et al. 2018, Heijman et al. 2020, Dobrev et al. 2023). Obese patients exhibit greater functional expression of the NLRP3 inflammasome complex in atrial myocardium compared with lean patients (Fender et al. 2020, Scott et al. 2021), and in obese mice, this leads to electrical remodeling, aberrant calcium handling, and increased susceptibility to AF (Scott et al. 2021). The contribution of PAR4-mediated thromboinflammation in the context of atrial cardiomyopathy has not been explored, nor have signaling interacting partners been identified. PAR4 is a promiscuous receptor that engages in cross-talks with other receptor systems by heterodimerization or transactivation (Gieseler et al. 2013, Lin et al. 2013). In pancreatic and hepatic tumor cell-lines, thrombin was some 20 years ago reported to rapidly transactivate the tyrosine kinase c-met, independently of the cognate ligand hepatocyte growth factor (HGF) (Fischer et al. 2004). The culprit receptor mediating this cross-talk was not clarified at the time, but the relatively high concentration of thrombin used (2 U/mL) suggests involvement of low-affinity PAR4 rather than the prototypical high-affinity PAR1. To date, thrombin/c-met cross talk has not been reported in the heart, nor is it known if such an interaction has functional significance that could be potentiated in the context of obesity.

## Methods

### Materials

Human α-thrombin was obtained from American Diagnostica GmbH (Pfungstadt, Germany); PAR1 activating peptide (PAR1-AP, TFLLRN-NH_2_) and PAR4-AP (AYPGKF-NH_2_) were purchased from Tocris/Biotechne, Wiesbaden, Germany; SGX-523 was from Selleckchem, Cologne, Germany. All other materials were purchased from Merck KGaA/Sigma-Aldrich (Taufkirchen, Germany) unless otherwise stated.

### Human right-atrial appendage samples

Right-atrial appendages (RAA) were obtained immediately prior to cardiopulmonary bypass from patients undergoing open-heart surgery for coronary bypass grafting or valve replacement. Biopsies were stored in Tyrode’s solution and taken to the laboratory being either frozen for later use or employed for atrial cardiomyocyte isolation by enzymatic digestion and bovine serum albumin (BSA)-gradient filtration. Detailed protocols have been published (Voigt et al. 2013, Heijman et al. 2020). In brief, atrial tissues are submerged in Ca^2+^-free buffer and cut into small chunks of approximately 1 mm^3^. Tissues are enzymatic digested with collagenase I and protease XXIV for 35 min, in the presence of 20 µM Ca^2+^, centrifuged at 95 × g for 10 min, and then re-suspended at room temperature in Ca^2+^-free Tyrode’s solution supplemented with 0.1% BSA. The cell suspensions are then layered over a 6% BSA gradient. Rod-shaped, striated cardiomyocytes were allowed to sediment to the bottom of the tubes over 45 min and directly lysed for protein isolation. The individual clinical characteristics of the cohort of *n*=6 obese (BMI ≥ 30) and *n*=6 control non-obese (BMI ≤ 25) patients have recently been published (Kleeschulte et al. 2024). Each patient gave written informed consent. The studies were approved by the Human Ethics Committee of the Medical Faculty of the University Duisburg-Essen (approval number AZ: 12-5268-BO) and were performed in accordance with the Declaration of Helsinki.

### Obese mouse atria

Archived atrial myocardium (pooled left and right atria) was obtained from male C57Bl/6J wild type and PAR4^-/-^ mice randomized in previous studies (Fender et al. 2020, Kleeschulte et al. 2024) at 8 weeks to receive standard chow or a high-fat diet (HFD, #S7200-E010, ssniff Spezialdiäten GmbH, Soest, Germany). Atria were collected after 8 weeks of feeding, at which time the wild-type HFD-fed mice displayed increased visceral adiposity, insulin resistance, glucose intolerance (Kleeschulte et al. 2024), and myocardial NLRP3 inflammasome activation (Fender et al. 2020) compared with chows. Each of these parameters was lower in HFD-fed PAR^-/-^ mice. Mice were housed at a 12-h light/dark cycle with *ad libitum* access to water and received humane care at all times. All animal experiments were performed in accordance with the ARRIVE and IMPROVE guidelines and the animal welfare guidelines of the University of Düsseldorf, with approval of the local authority (Landesamt für Natur, Umwelt und Verbraucherschutz, LANUV Nordrhein-Westfalen, Bezirksregierung Düsseldorf, Az. 81-02.04.2017.A458 and 81-02.04.2022.A297).

### HL-1 atrial cardiomyocytes

HL-1 cells and Claycomb HL-1 culture medium were purchased from Sigma-Aldrich; cells were maintained under standard culture conditions and studied at passage 7–10. Study drugs included thrombin (1 U/mL), PAR1-AP (50 µM), PAR4-AP (100 µM), or appropriate vehicle control; the c-met inhibitor SGX-523 (1 µM) was pre-incubated 15 min prior to addition of PAR4-AP. Stimulation was performed in a reverse manner (time-matched protocol) with all cell supernatants and monolayers collected at the same final end-point. Study drugs or vehicle were added at appropriate intervals before sample collection, as aliquots of 100–1000× stock solutions of study drug or vehicle supplied to all wells.

### Immunoblot

Protein lysates were prepared from cells and frozen tissue and immunoblotted using the LI-COR Odyssey platform as described (Mann et al. 2024). Primary antibodies against PAR1, caspase-1, and total CaMKIIδ were from Santa Cruz Biotechnology; PAR4 and IL-1β antibodies are from Abcam, Berlin, Germany; phospho-Thr287-CaMKII, phospho-Ser473-AKT1, phospho-Ser2481-mTOR, total mTOR, phospho-Thr-172-AMPKα, total AMPKα, and phospho-Tyr1234/1235-c-met antibodies were all from Cell Signaling Technology, Danvers MA, USA; the antibodies against total c-met, total AKT1, and the housekeeper γ-tubulin were from Thermo Fisher Scientific, Waltham, MA, USA. All primary and infrared-coupled secondary antibodies (LI-COR Biosciences, Bad Homburg, Germany) were diluted 1:1000. Band densities of PAR1 and PAR4 in cardiomyocytes were normalized to Ponceau, and band densities of phosphorylated and unphosphorylated proteins were each normalized to γ-tubulin. For caspase-1 and IL-1β, the ratio of the cleaved form to its uncleaved precursor is depicted. Uncut blot are provided in the data supplement 2, and yellow boxes indicate the sections shown in the main figures.

### Quantitative real-time PCR

RNA was extracted using the RNeasy Mini Kit and reverse transcribed to cDNA using the QuantiTect® Reverse Transcription Kit (both from Qiagen, Erkrath, Germany) as instructed by the manufacturer. The StepOnePlus™ Real-Time PCR System (Life Technologies, Singapore, Singapore) was used to measure mRNA levels of *Met*, *Acta2*, and the three housekeeper genes *Gata4*, *Hmbs*, and *B2m*, using Quantitect Validated Primer Assays from Qiagen and Platinum® SYBR® Green qPCR SuperMix-UDG (Life Technologies, USA. Target mRNA abundance was normalized to each the three housekeeping genes using the ΔΔCt method and meaned.

### ELISA and fluorometric kits

Frozen tissue and cell supernatants were assessed using the following commercial kits as instructed by the manufacturer: Human IL-1beta Uncoated ELISA Kit (Thermo Fisher), Mouse IL-1beta Colorimetric ELISA Kit (Biomatik, via Biozol, Eching, Germany), and OxiSelect Myeloperoxidase Chlorination Activity Assay Kit (BioCat GmbH, Heidelberg, Germany).

### Statistical analysis

Data are depicted as fold of the respective control or absolute values as appropriate. Statistical testing of two groups utilized the unpaired *t*-test; with more groups, one-way analysis of variance (Kruskal-Wallis) was applied with Dunn’s multiple comparison procedure as appropriate, using GraphPad Prism. *P*<0.05 was accepted as statistically significant.

## Results

### Increased thrombin receptor expression in atrial cardiomyocytes of obese patients

**Fig. 1:**
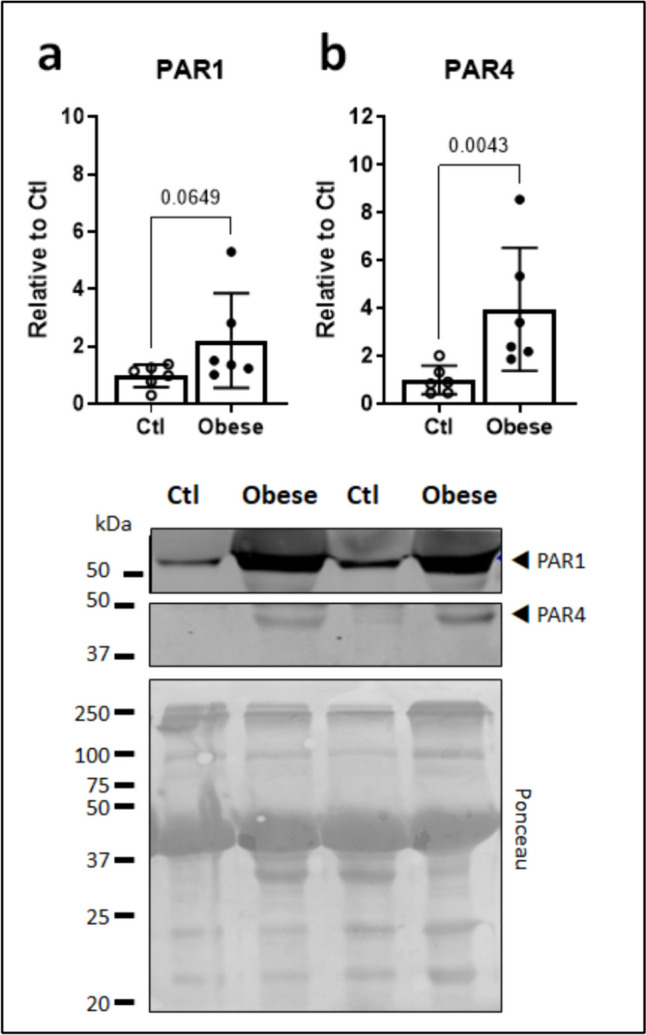
Thrombin receptor expression in human atrial cardiomyocytes. **a** PAR1 and **b** PAR4 protein expression, normalized to Ponceau staining, in cardiomyocyte-enriched fraction of right atrial appendages from obese versus non-obese control (Ctl) patients undergoing cardiac surgery. All *n*=6

PAR1 and PAR4 expression was assessed in cardiomyocyte-enriched fractions of right atrial appendages obtained from obese versus non-obese patients undergoing cardiac surgery. PAR1 was abundant in non-obese atrial cardiomyocytes with a twofold higher expression level in the obese group (Fig. 1a). PAR4 expression was very low in non-obese atrial cardiomyocytes but fourfold higher and significantly so in the obese group (Fig. 1b).

### PAR4 activates pro-inflammatory signaling platforms in HL-1 atrial cardiomyocytes

**Fig. 2:**
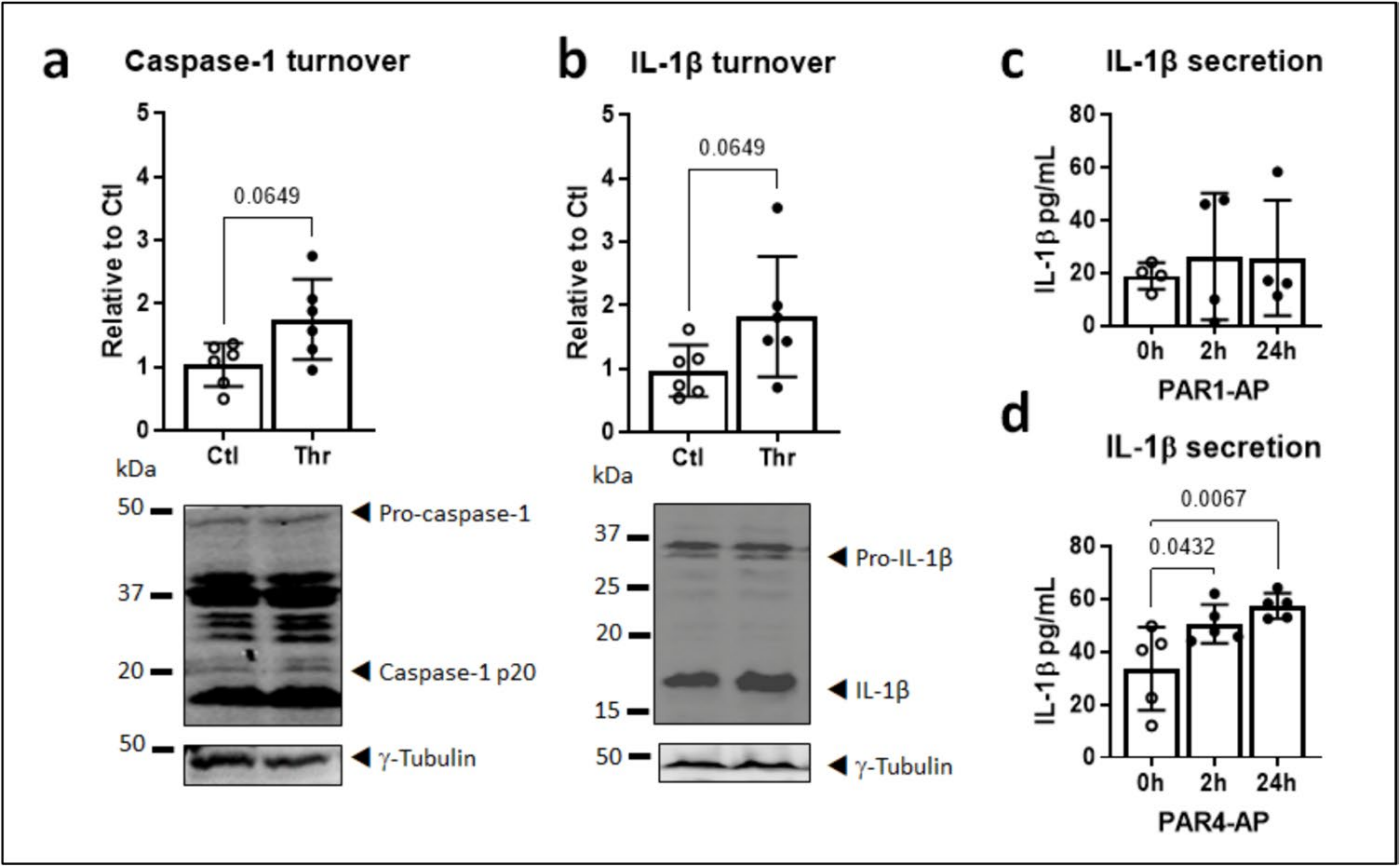
Thrombin stimulates IL-1β production in HL-1 atrial cardiomyocytes. **a** Turnover of caspase-1 and **b** IL-1β, indicating auto-proteolytic activation and maturation, respectively, in HL-1 cardiomyocytes stimulated with thrombin or vehicle for 24 h. Turnover was determined as the ratio of cleaved (active) to uncleaved (precursor) proteins. **c** Secreted IL-1β in the conditioned medium of HL-1 cells stimulated for the indicated times with PAR1 or **d** PAR4-activating peptides (AP). All *n*=4–6 as indicated

Thrombin elicits IL-1β production in ventricular fibroblasts (Kleeschulte et al. 2018, Fender et al. 2020). To verify that thrombin also provokes NLRP3 inflammasome-dependent IL-1β release in atrial cardiomyocytes, HL-1 cells were stimulated with 1 U/mL thrombin for 24 h. Caspase-1 turnover, a measure of proteolytic auto-activation, was determined as the ratio of cleaved caspase-1 (p20) to the precursor pro-caspase-1; similarly, IL-1β turnover (ratio IL-1β/pro-IL-1β) was taken to indicate IL-1β maturation (Fender et al. 2020). Both parameters were approximately twofold higher in thrombin-treated versus control cardiomyocytes (Fig. 2a, b). Basal release of IL-1β to the extracellular medium was significantly increased within 2 h upon addition of a selective PAR4-activating peptide (AP) but not by the PAR1-AP (Fig. 2c, d).

**Fig. 3:**
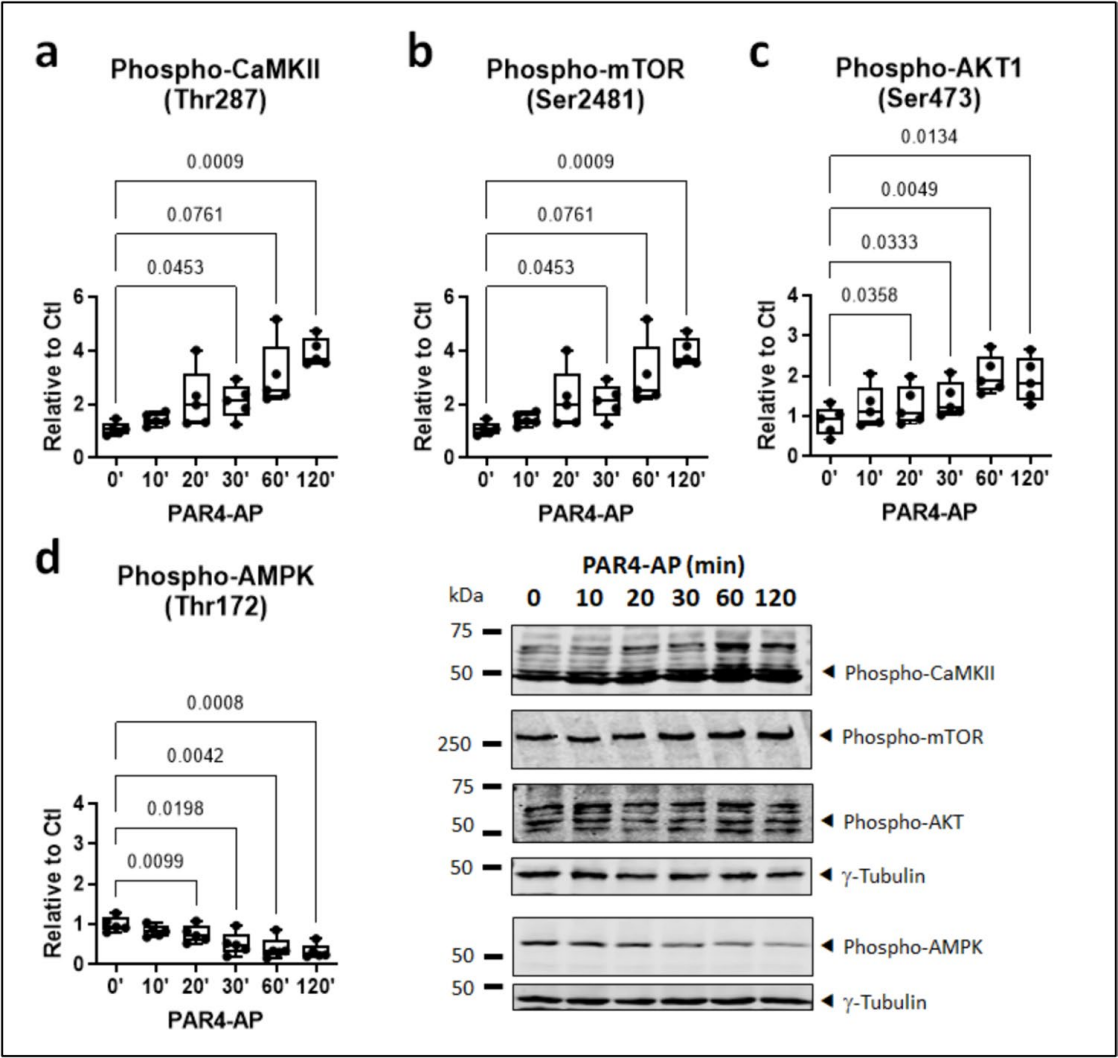
PAR4 regulates inflammation- and arrhythmia-associated kinases in HL-1 atrial cardiomyocytes. **a** Phosphorylated CaMKII, **b** mTOR, **c** AKT, and **d** AMPK, in HL-1 cells stimulated with PAR4-activating peptide (AP) for the indicated times. All *n*=5, all normalized to γ-tubulin

**Fig. 4:**
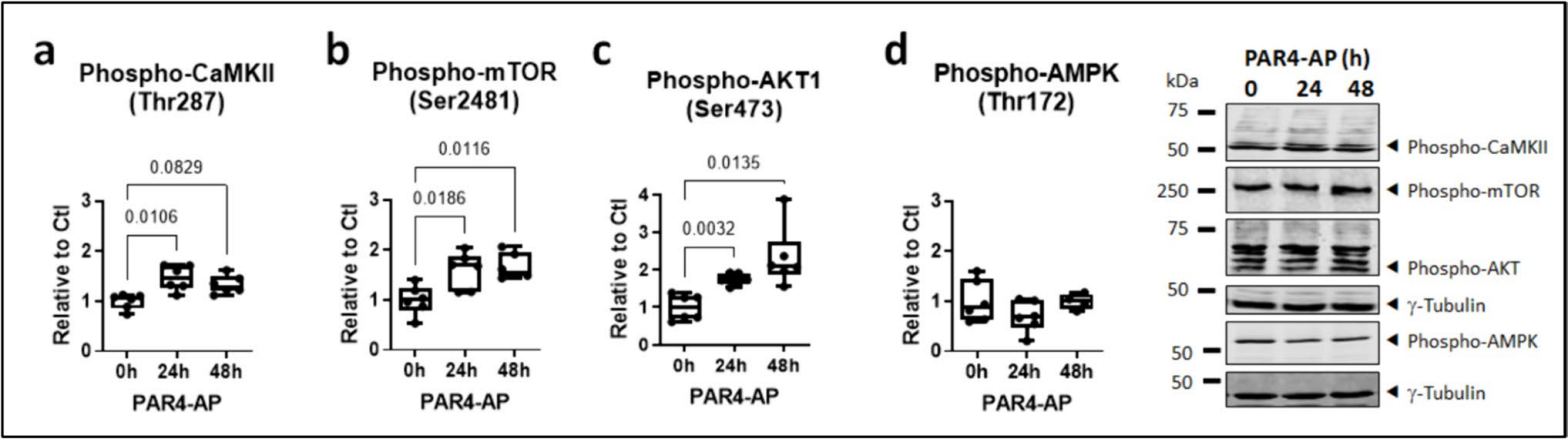
Sustained phosphorylation of inflammation- and arrhythmia-associated kinases in response to PAR4 activation in HL-1 atrial cardiomyocytes. **a** Phosphorylated CaMKII, **b** mTOR, **c** AKT, and **d** AMPK, HL-1 cells stimulated with PAR4-AP for the indicated times. All *n*=6, all normalized to γ-tubulin

Since NLRP3 inflammasome activation is linked with signaling through CaMKII (Heijman et al. 2020) and the AKT/mTOR pathway (Lu et al. 2024), phosphorylation of these kinases was specifically assessed in HL-1 cells stimulated with PAR4-AP. PAR4 activation resulted in a rapid and progressive increase in the phosphorylation of CaMKII, mTOR and AKT at the respective activation sites, Thr287, Ser2481, and Ser473 (Fig. 3a–c). By contrast, phosphorylation of the negative mTOR regulating kinase AMPK at Thr172 was suppressed (Fig. 3d). The regulatory impact of PAR4 activation on phosphorylated CaMKII, mTOR, and AKT was largely sustained over 24–48 h (Fig. 4). No effect was noted in terms of total, unphosphorylated, kinase levels over the entire time-course (data supplement 1, Figures S1, S2), with the exception of total AMPK, which was modestly increased over 48 h of incubation with PAR-4AP (data supplement 1, Figure S2).

### PAR4 signaling in atrial cardiomyocytes partially requires c-met cross-activation

**Fig. 5:**
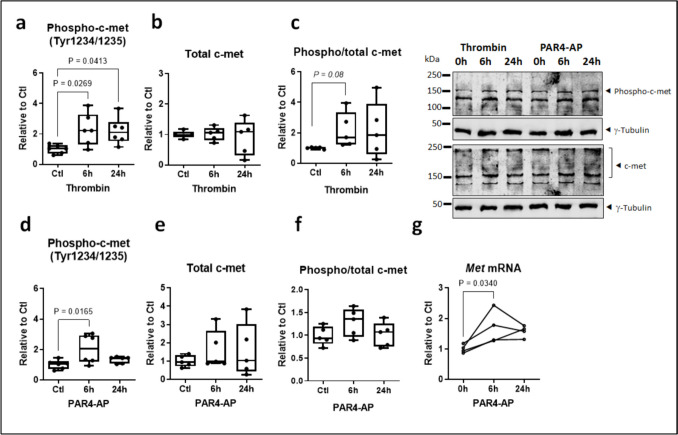
PAR4 regulates c-met in HL-1 atrial cardiomyocytes. **a** Phosphorylated and **b** total c-met, both normalized to γ-tubulin, and **c** proportional c-met phosphorylation in HL-1 cells stimulated with thrombin or **d**–**e** PAR4-activating peptide (AP) for the indicated times (all *n*=5–6). **e** *Met* mRNA (*n*=4) in HL-1 atrial cardiomyocytes stimulated with PAR4-AP for the indicated times

**Fig. 6:**
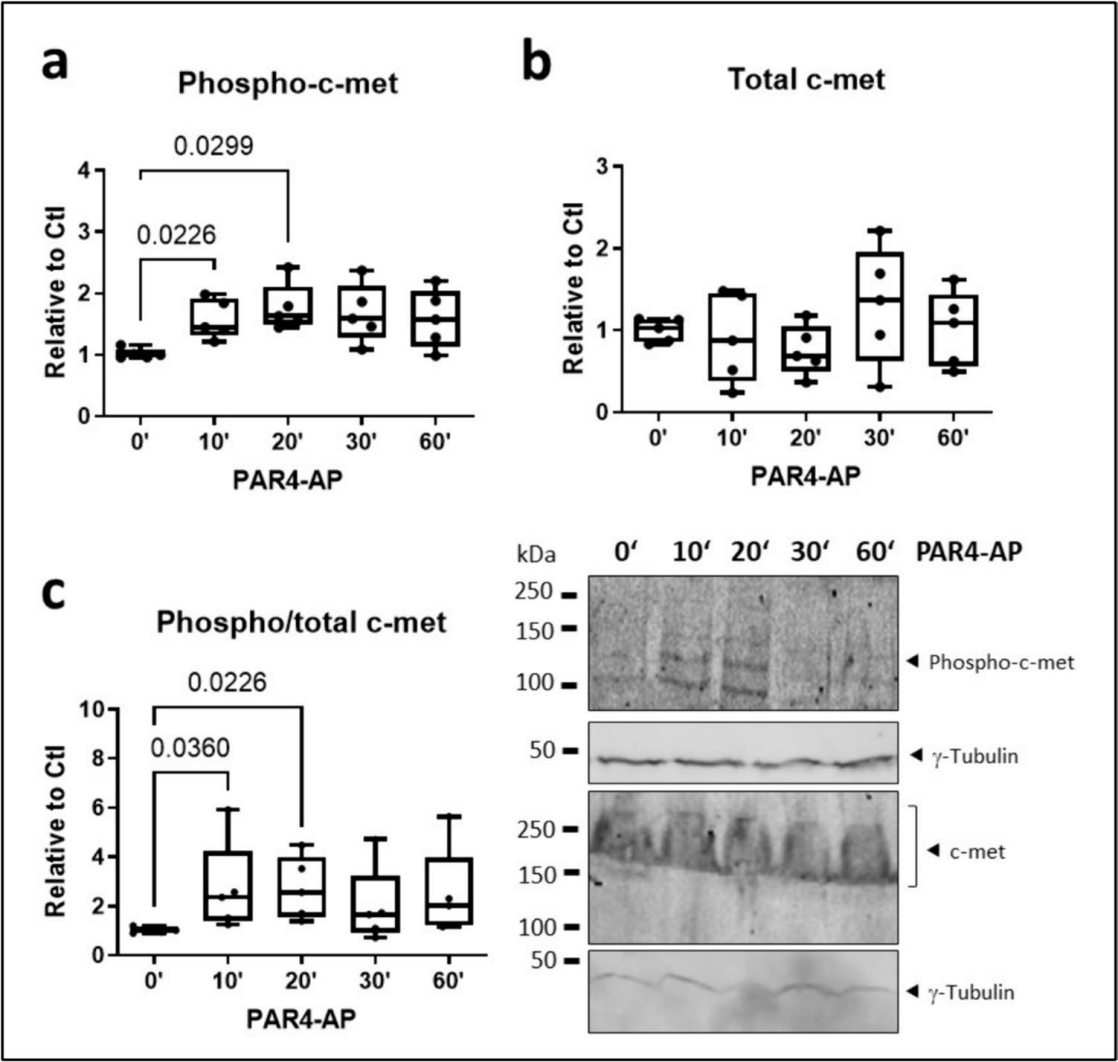
PAR4 acutely transactivates c-met in HL-1 atrial cardiomyocytes. **a** Phosphorylated and **b** total c-met, both normalized to γ-tubulin, and **c** proportional c-met phosphorylation in HL-1 cells stimulated PAR4-activating peptide (AP) for the indicated times, all *n*=6

Thrombin has been reported to cross-activate c-met in some cancer cell-lines (Fischer et al. 2004). In HL-1 atrial cardiomyocytes, thrombin elicited a sustained increase in phosphorylated c-met over 6–24 h (Fig. 5a), while total c-met levels were largely unaltered (Fig. 5b). The proportional phosphorylation of c-met (ratio phospho/total c-met) was significantly increased at 6 h but no longer at 24 h (Fig. 5c). Over the same time-course, PAR4-AP increased phosphorylated c-met markedly and also tended to elevate total c-met, so that the modest increase in proportional c-met phosphorylation was not significant (Fig. 5d–f). Application of PAR4-AP also increased levels of *Met* transcript in HL-1 cells (Fig. 5d, e). PAR4-mediated c-met phosphorylation occurred rapidly, within 10–20 min of PAR4 activation (Fig. 6a). Total c-met remained unchanged over the acute time-course; the proportional c-met phosphorylation was also significant over 10–20 min of PAr4 activation (Fig. 6b,c).

**Fig. 7:**
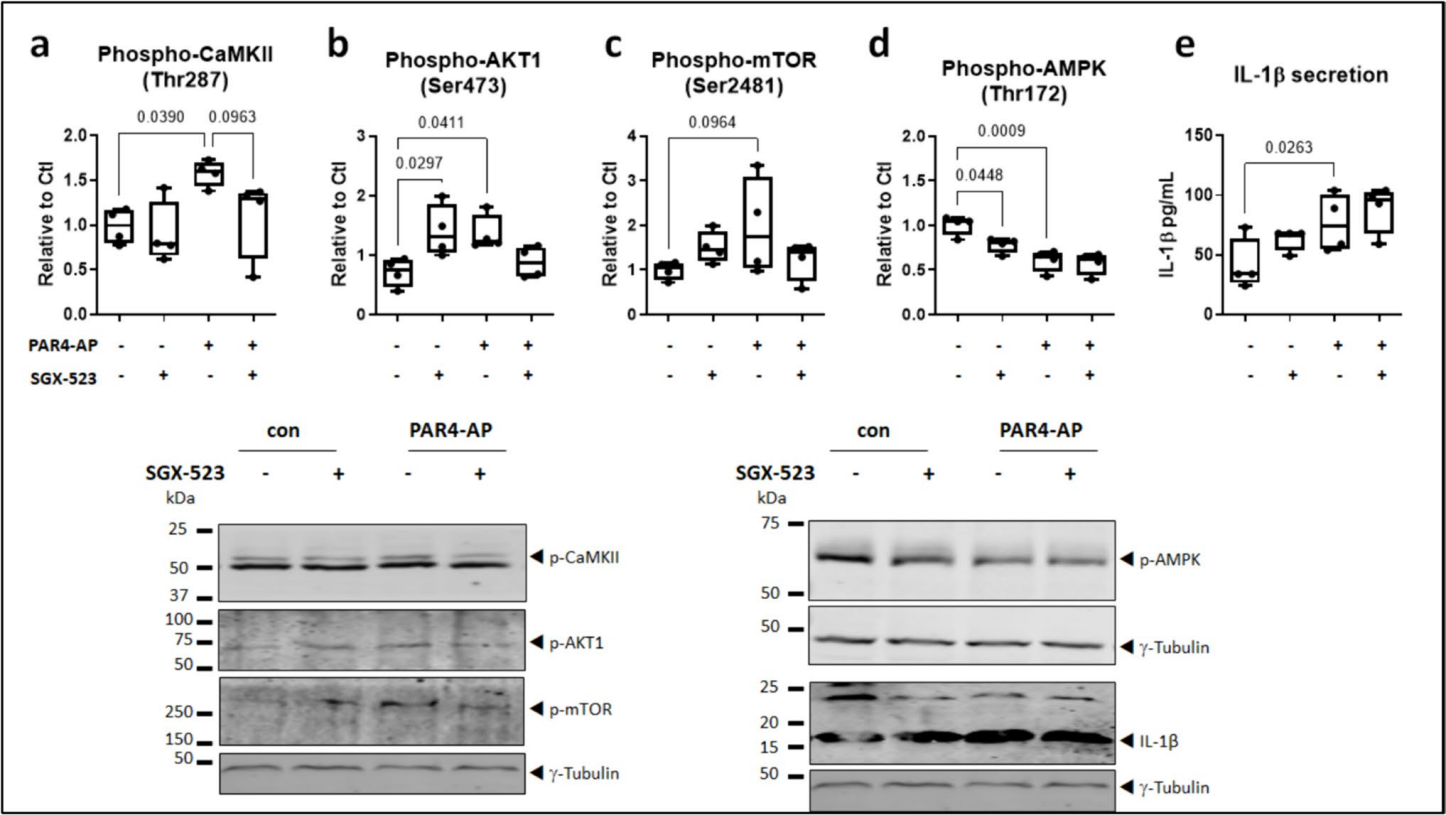
Transactivated c-met partially contributes to PAR4-evoked signaling but not IL-1 β production. **a** Phosphorylated CaMKII, **b** AKT, **c** mTOR, and **d** AMPK in HL-1 cells pre-incubated with the c-met inhibitor SGX-523 for 15 min prior to stimulation with PAR4-activating peptide (AP) for 24 h. **e** IL-1β secretion by HL-1 cells treated with SGX-523 and then PAR4-AP for 24 h. All *n*=4, all normalized to γ-tubulin.

To dissect the potential involvement of cross-activated c-met in atrial cardiomyocyte signaling downstream of activated PAR4, HL-1 cells were pre-incubated for 15 min with the selective c-met inhibitor SGX-523 prior to addition of PAR4-AP for 24 h. SGX-523 abrogated the stimulatory effect of PAR4-AP on phosphorylation of CaMKII, AKT and mTOR (Fig. 7a–c) but did not modify the suppression of AMPK phosphorylation nor the release of IL-1β in response to PAR4-AP (Fig. 7d–f). Total unphosphorylated CamKII, AKT, and mTOR were unaffected by the treatments, but total AMPK showed a marginal yet significant increase with PAR4-AP and normalization in combination with the inhibitor (data supplement 1, Figure S3).

### Obesity in vivo largely reproduces the features of PAR4-stimulated atrial cardiomyocytes

**Fig. 8:**
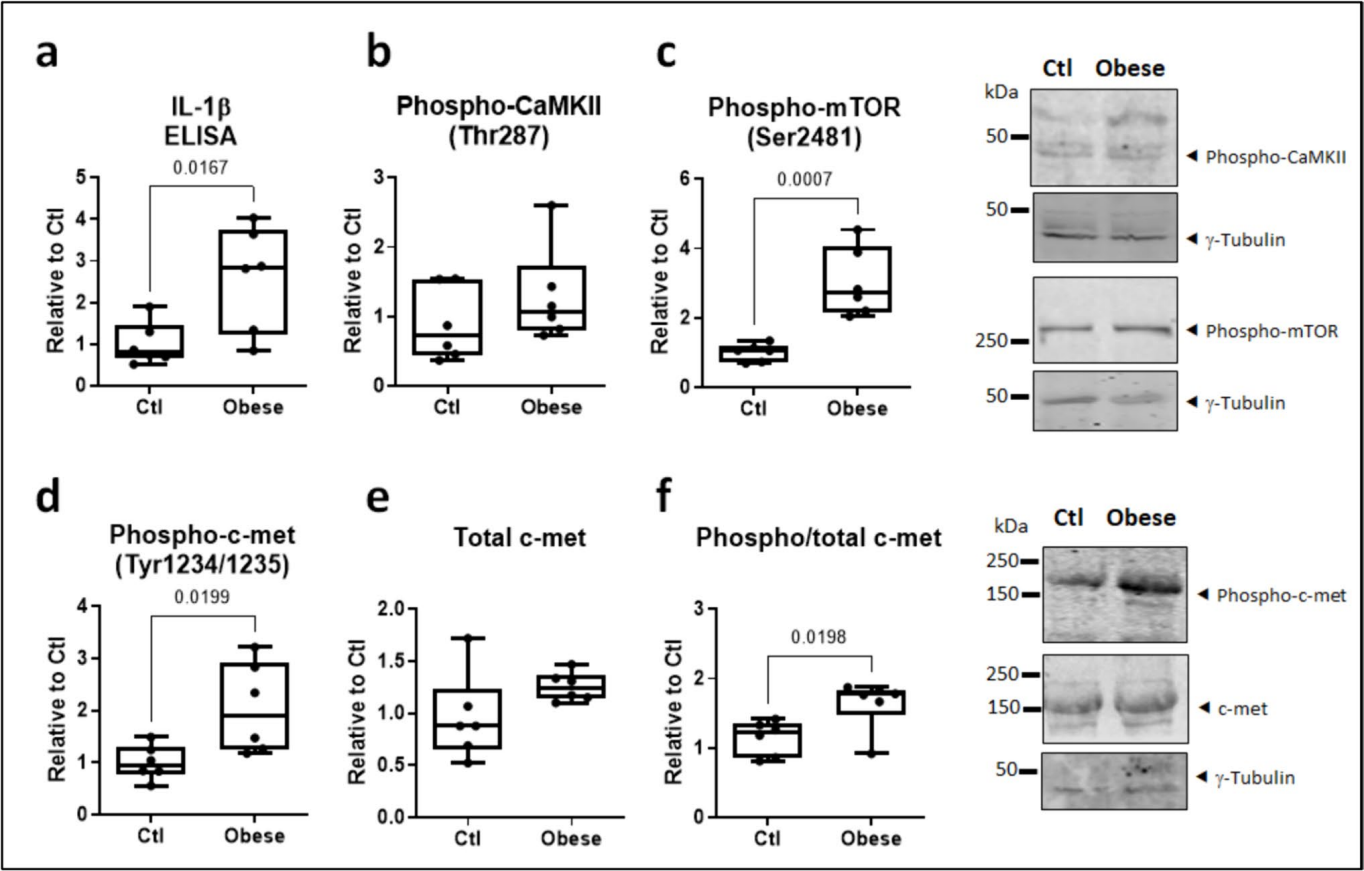
Clinical obesity increases atrial IL-1β and phosphorylated c-met and mTOR. **a** IL-1β content determined by ELISA, **b** phosphorylated CaMKII, **c** phosphorylated mTOR, **d** phosphorylated c-met, and **e** total c-met determined by immunoblotting (all normalized to γ-tubulin), in right atrial appendage lysates from obese versus non-obese control (Ctl) patients undergoing cardiac surgery. **f** Proportional c-met phosphorylation (ratio phospho/total c-met). All *n*=6

Increased PAR4 abundance coincides with an activated NLRP3 inflammasome in atrial myocardium of obese diabetic patients (Fender et al. 2020). In this study, obese human atria were confirmed to contain more IL-1β than non-obese biopsies (Fig. 8a). Obesity did not significantly affect abundance of phosphorylated (Fig. 8b) or total CaMKII (data supplement 1, Figure S4) but did increase abundance of phospho-mTOR and phospho-c-met (Fig. 8c, d). Total atrial mTOR was unaffected (data supplement 1, Figure S4), while total c-met was only marginally higher in the obese cohort; accordingly, proportional c-met phosphorylation remained significant (Fig. 8e, f)

**Fig. 9:**
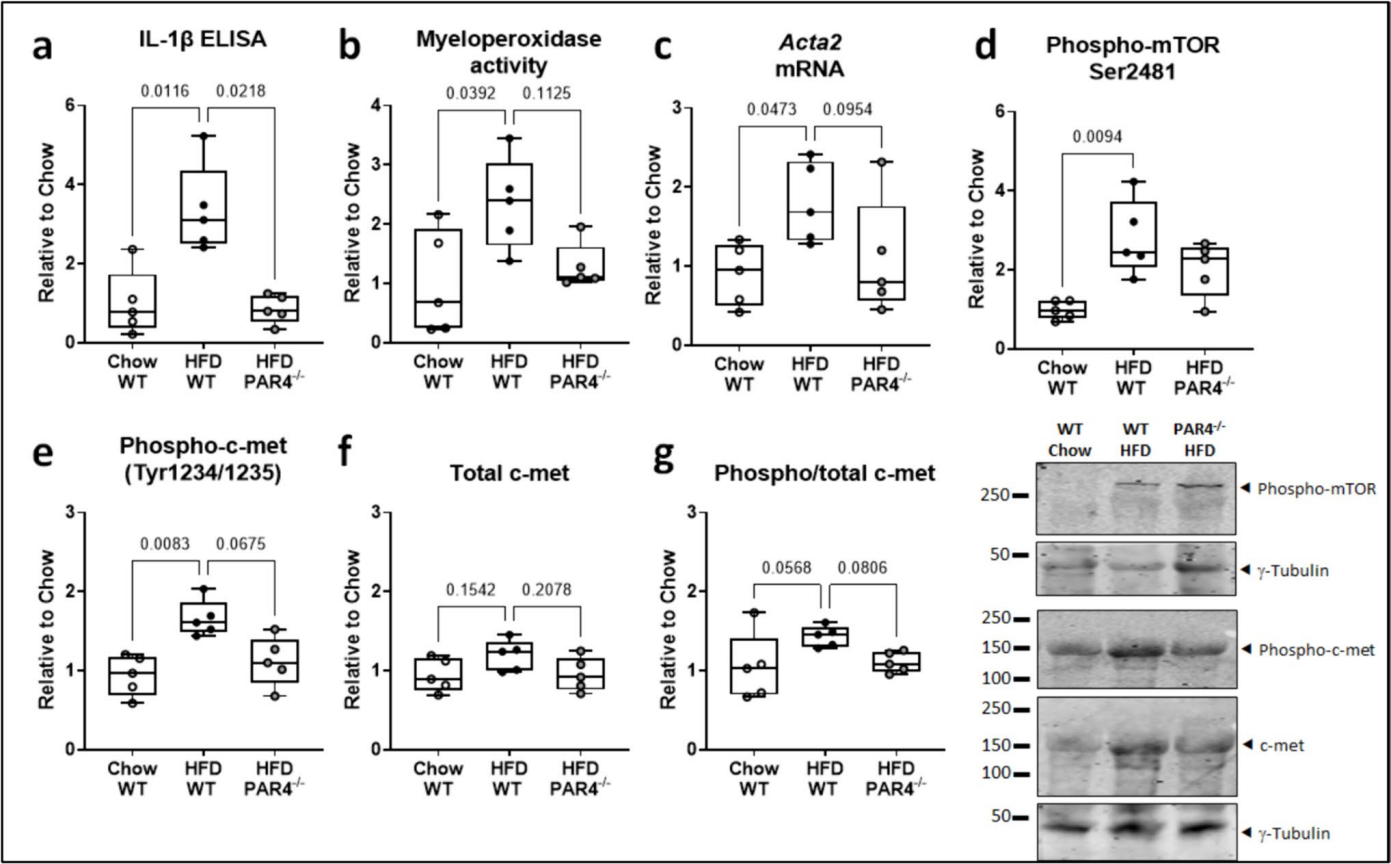
High-fat diet (HFD) increases atrial IL-1β and phosphorylated c-met and mTOR via PAR4 *in vivo*. **a** IL-1β content determined by ELISA, **b** myeloperoxidase activity determined by chlorination assay, **c** *Acta2* mRNA determined relative to ribosomal 18S, **d** phosphorylated mTOR, **e** phosphorylated c-met, and **f** total c-met, determined by immunoblotting relative to γ-tubulin, in atrial lysates from wild type (WT) or PAR4^-/-^ mice fed chow or high-fat diet (HFD) for 8 weeks. **g** Proportional atrial c-met phosphorylation (ratio phospho/total c-met). All *n*=6

Atria of mice fed a high-fat diet (HFD also contained more IL-1β than atria from chow-fed mice), and this was abrogated in the PAR4^-/-^ mice (Fig. 9a). Myeloperoxidase activity, *Acta2* mRNA, and phosphorylated mTOR showed the same trend, increasing with HFD and reducing with PAR4 deletion (Fig. 9b–d.) Total mTOR remained unaffected (data supplement 1, Figure S5). Phosphorylated c-met was also significantly higher in the HFD-fed mice, but total c-met only marginally so, and accordingly, the proportional c-met phosphorylation was near-significant (*P*=0.0568). This effect was largely absent in the PAR4-deficient mice (Fig. 8e–g).

## Discussion

**Fig. 10:**
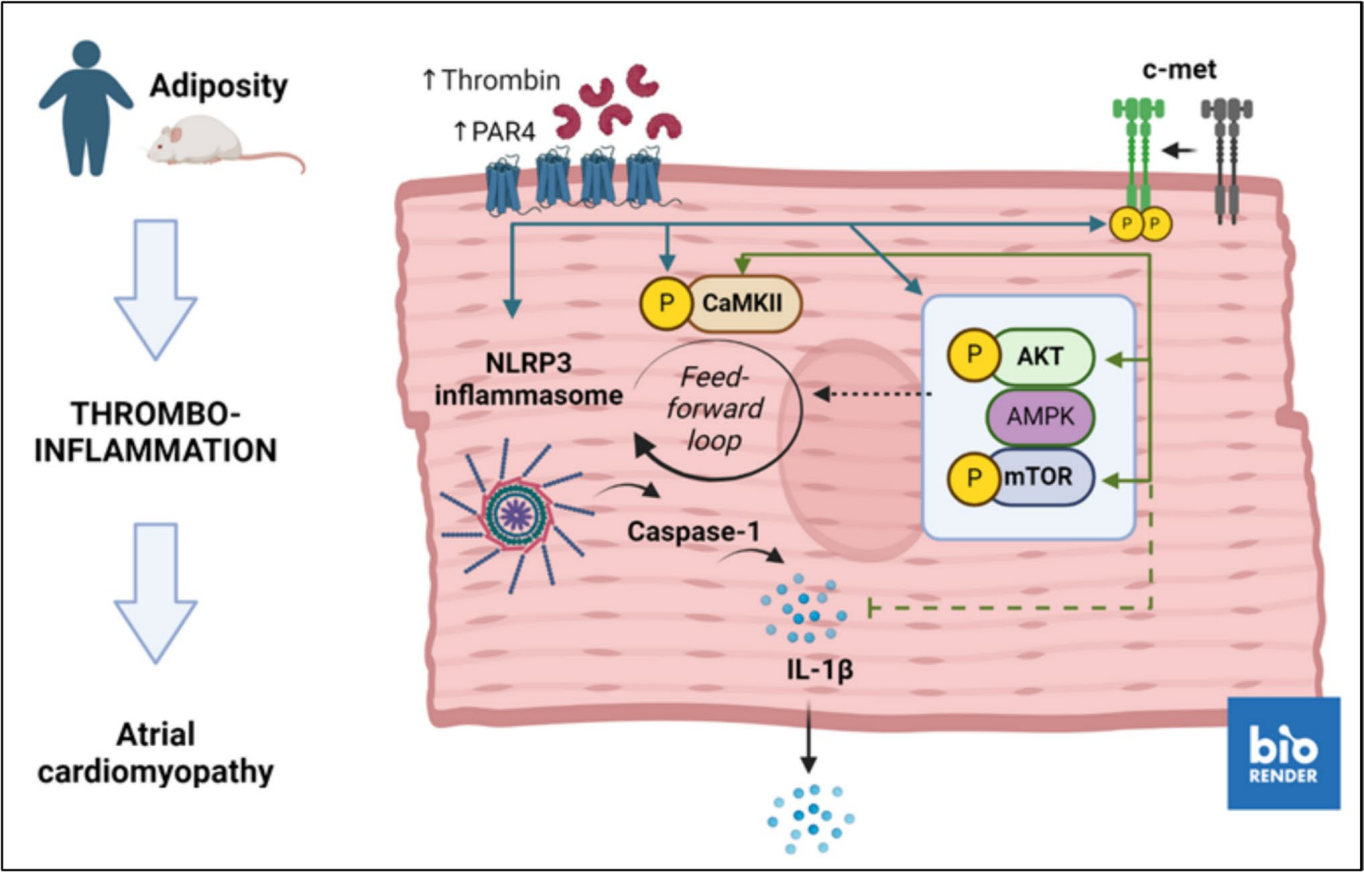
Graphical abstract. Thrombin receptor PAR4 is upregulated in atria of obese humans and mice. Activated PAR4 promotes NLRP3 inflammasome activation, culminating in IL-1β maturation and release, a key driver of inflammatory atrial cardiomyopathy. In atrial cardiomyocytes, PAR4 mediates the phosphorylation of CaMKII and mTOR while suppressing AMPK phosphorylation. Concurrent ligand-independent transactivation of c-met contributes to PAR4-mediated activation of CAMKII/mTOR, but is not involved in AMPK inhibition or production of IL-1β. Selective targeting of PAR4 and c-met may provide a therapeutic perspective in the context of obesity-driven thromboinflammation and atrial cardiomyopathy

Our major new findings are that (i) PAR4 is functionally expressed in atrial cardiomyocytes and mediates thrombin-stimulated IL-1β production; (ii) ligand-independent transactivation of c-met partially contributes to PAR4-evoked signaling, but not IL-1β production; and (iii) this cross-talk is likely to be active also *in vivo* in the context of metabolic atrial cardiomyopathy and thromboinflammation (Fig. 10).

Obesity is an inflammatory and hypercoagulant state (Malandish and Gulati 2023) characterized by chronically elevated thrombin levels (Chitongo et al. 2017, Bertaggia Calderara et al. 2020) and an overactive NLRP3 inflammasome (Bauer et al. 2023, Cho et al. 2023). This constellation (Vahdat 2022), conceptually captured by the term “thromboinflammation,” likely contributes to the cardiac sequelae of visceral adiposity, such as atrial cardiomyopathy and AF (Scott et al. 2021, Gawałko et al. 2023). PAR-thrombin receptors may therefore be an underappreciated driver of cardiometabolic diseases. In human and murine ventricular fibroblasts, enforced upregulation of PAR4 by hyperglycemic culture conditions increased IL-1β production in response to thrombin, which could be largely prevented by selective PAR4 inhibition (Kleeschulte et al. 2018, Fender et al. 2020). We also found that PAR4 is over-expressed in the atrial myocardium of obese diabetic patients and correlates with an activated NLRP3 inflammasome (Fender et al. 2020). However, a thrombin/PAR4/NLRP3 inflammasome axis has not been demonstrated in atrial cardiomyocytes, from which pro-arrythmic inflammatory signaling critically originates (Yao et al. 2018, Dobrev et al. 2023).

Over 15 years ago, functional expression of the prototypical thrombin receptor PAR1 was indirectly demonstrated in primary human atrial cardiomyocytes, in which the PAR1/PAR2 dual agonist SFFLRN mimicked the stimulatory effect of thrombin on persistent sodium current, while PAR1-selective antagonists blocked it (Pinet et al. 2008). More recently, HL-1 atrial cardiomyocytes were shown to express both PAR1 and the low-affinity thrombin receptor PAR4 (Ruf et al. 2023). We have now validated the expression of both receptors in primary human atrial cardiomyocytes and, to our knowledge, demonstrated for the first time a markedly increased abundance in cardiomyocytes from obese compared with non-obese patients undergoing cardiac surgery. The relative difference in expression level was approximately twofold for PAR1 and fourfold for PAR4, which, however, was only sparely detected in the non-obese group. This reflects our previous observations in whole atrial lysates from obese diabetic versus control patients (Fender et al. 2020) and is consistent with the role of PAR1 as a ubiquitous and constitutively expressed receptor, whereas PAR4 is more dynamically upregulated in response to inflammatory and diabetogenic stress (Dangwal et al. 2011, Pavic et al. 2014, Kleeschulte et al. 2018, Kleeschulte et al. 2024).

The immortalized mouse cardiac muscle cell-line HL-1 reproduces the phenotypic characteristics of adult primary atrial cardiomyocytes (Claycomb et al. 1998) and provides a suitable *in vitro* model system to study the mechanisms of cardiometabolic disease (Wong et al. 2021). In these cells, thrombin increased the turnover of auto-activated caspase-1 from the precursor pro-caspase-1, along with maturation of IL-1β. This confirms the ability of thrombin to activate the canonical NLRP3 inflammasome in atrial cardiomyocytes, as we have seen in human ventricular fibroblasts (Fender et al. 2020). The PAR1-selective activating peptide (AP) did not significantly increase IL-1β secretion in our hands, whereas PAR4-AP augmented basal IL-1β release in a rapid and sustained manner. This suggests that PAR4, rather than PAR1, mediates NLRP3-dependent thromboinflammation in atrial cardiomyocytes. PAR4 activation elicited a rapid and sustained phosphorylation of CaMKII at Thr287, which is causally linked with NLRP3 inflammasome activation in atrial cardiomyocytes and the vulnerable AF substrate (Heijman et al. 2020), and of mTOR and AKT, which are functionally linked with CaMKII activation (Dong et al. 2019, Fan et al. 2021), as well as NLRP3 inflammasome activity and autophagic redox stress in atrial cardiomyocytes (Bairashevskaia et al. 2022, Lu et al. 2024). Total protein levels of unphosphorylated CamKII, AKT, and mTOR were not changed in response to PAR4 activation over the entire time-course studied. Phosphorylation of AMPK was by contrast progressively reduced during exposure to PAR4-AP, while total AMPK levels increased, albeit modestly yet reaching significance after 48 h. AMPK has recently emerged as a critical suppressor of inflammatory signaling in AF (Brown et al. 2021, Ozcan et al. 2021, Su et al. 2022). Its sustained inhibition may therefore represent a mechanism by which thrombin/PAR4 facilitate the shift toward inflammatory versus anti-inflammatory states and potentially contribute to atrial cardiomyopathy and AF.

PAR4 signaling frequently engages other receptor systems by heterodimerization or transactivation (Gieseler et al. 2013, Lin et al. 2013). This aspect of PAR pharmacology has to date not been explored in the context of cardiometabolic disease or atrial cardiomyopathy. Twenty years ago, thrombin was suggested to transactivate c-met, the tyrosine kinase receptor for HGF, in a ligand-independent manner (Fischer et al. 2004). Since then, this phenomenon has not been validated in other cells, nor has the culprit receptor involved identified. The concentration of thrombin used in that study, 2 U/mL, is highly in excess even of the level required to activate low-affinity PAR4. Here, we exposed HL-1 atrial cardiomyocytes to 1 U/mL of thrombin and observed a sustained increase in absolute and proportional c-met phosphorylation at the activation site Tyr1234/1235; total c-met was largely unchanged at both the 6 h and 24 h time-point studied. Activation of thrombin receptor PAR4 also increased phospho-c-met levels in HL-1 cells; both absolute and proportional c-met phosphorylation occurred rapidly and significantly within 10–20 of stimulation. At later time-points (6–24 h), concurrent transcriptional upregulation of c-met gene and protein expression largely abrogated proportional c-met phosphorylation. However, at least for the acute setting, we could validate HGF-independent transactivation of c-met by thrombin and identify PAR4 as the mediating receptor.

To assess the functional relevance of this cross-talk, HL-1 cells were pre-treated with the selective c-met inhibitor SGX-523 prior to stimulation with the PAR4 agonist for 24 h. In the presence of the inhibitor, PAR4-AP failed to significantly increase phosphorylation of its target kinases CaMKII, AKT, and mTOR. PAR4-mediated suppression of AMPK phosphorylation was by contrast preserved, as were IL-1β maturation and secretion. Thus, c-met transactivation contributes partially to PAR4-evoked signaling in atrial cardiomyocytes, but IL-1β production is directly supported by PAR4, independently of c-met. The c-met inhibitor *per se* was noted to increase AKT phosphorylation and to a lesser extent, mTOR phosphorylation, along with suppression of phosphorylated AMPK. This implicates some degree of constitutive, inhibitory c-met signaling that is unmasked with SGX-523, while cross-activation by PAR4 shifts c-met toward stimulatory signaling. This concept needs to be verified in atrial cells, but would be consistent both with the constitutive activation of c-met reported in certain tumor cells (Rusciano et al. 1996, Dai and Siemann 2012) and with the context-dependent protective versus adverse duality of c-met function in cardiac and metastatic diseases (Leo et al. 2011, Centuori and Bauman 2022, Jin et al. 2024).

To translate our *in vitro* findings to the clinical setting, we obtained atrial whole tissue lysates from obese versus non-obese patients undergoing cardiac surgery. IL-1β levels, determined by ELISA, were markedly higher in the obese group, confirming our recent report that obesity promotes atrial NLRP3 inflammasome activation (Scott et al. 2021). Unexpectedly, CaMKII phosphorylation, which we recently linked with an activated atrial NLRP3 inflammasome in patients prone to develop new-onset post-operative AF (Heijman et al. 2020), was not augmented. Increased IL-1β levels did however coincide with increased phosphorylation of both c-met and mTOR, reflecting our *in vitro* observations in PAR4-stimulated cardiomyocytes.

To identify a causal link between PAR4, atrial inflammatory signaling, and c-met, we examined atria from mice fed a HFD for 8 weeks. We previously found that HFD-fed mice display PAR4 overexpression, visceral adiposity, and myocardial and adipose tissue inflammation (Kleeschulte et al. 2018, Fender et al. 2020, Kleeschulte et al. 2024). Mirroring what we saw in obese human atria, atria from HFD-fed mice contained higher levels of IL-1β and phospho-mTOR, together with increased myeloperoxidase activity, which derives mainly from neutrophils and to a lesser extent monocytes, as well as the *Acta2* transcript which encodes alpha smooth muscle actin. Altogether, the HFD-fed mouse atria displays a pro-inflammatory and pro-fibrotic state, which was largely abrogated in mice with genetic PAR4 deletion. At the same time, HFD led to an increased abundance of phosphorylated c-met and a more moderate rise in total c-met protein, which again were blunted in the PAR4^-/-^ mice. Overall, a setting of increased PAR4 expression and activation appears to go in hand with augmented c-met expression and activation. While this is not evidence for a direct causal link between PAR4/c-met and atrial inflammatory signaling *in vivo*, the association is to our mind strong enough to warrant further study.

One major limitation of our study is that pooled left and right mouse atrial tissues were assessed in order to obtain sufficient material. Considering that left and right atria show distinct functional, genotypic, and phenotypic features (Kahr et al. 2011, Hsu et al. 2012, Butova et al. 2023, Li et al. 2023), ongoing and future studies will need assess these tissues separately. We also unfortunately omitted analysis of chow-fed PAR4^-/-^ mice. In a type 1-diabetes mouse model with carotid artery injury, PAR4 deletion reduced the aggravated neointima formation and macrophage infiltration seen in diabetic animals, but did not affect the more modest vascular remodeling and inflammation in non-diabetic animals (Pavic et al. 2014). In isolated vascular smooth muscle cells also, PAR4 siRNA had no effect on control cells, but did blunt the stronger thrombin responses cells previously challenged with high glucose (Dangwal et al. 2011). PAR4 expression is generally low in most non-platelet cells, so we expect PAR4 deletion to manifests phenotypically in settings of oxidative or metabolic stress, when PAR4 is upregulated (Fender et al. 2017).

Taken together, we demonstrate a functional PAR4/c-met cross-talk in atrial cardiomyocytes that contributes in part to local thrombo-inflammatory signaling. The stimulatory effect of PAR4 on NLRP3 inflammasome-dependent IL-1β production appears to be largely independent of c-met. The potential for inter-receptor communication may be particularly important in the context of obesity. Selective small-molecule antagonists of PAR4 (Merali et al. 2023, Nash et al. 2024) and c-met (Kudo et al. 2020, Wu et al. 2023) which are currently undergoing clinical evaluation therefore offer new therapeutic perspectives for the treatment of obesity-related atrial cardiomyopathy and its sequelae.

## Supplementary Information

Below is the link to the electronic supplementary material.

Supplementary file1 (PDF 722 KB)

Supplementary file2 (PDF 1026 KB)

## Data Availability

Data and materials may be available from the corresponding author at reasonable request.

## References

[CR1] Bairashevskaia AV, Belogubova SY, Kondratiuk MR, Rudnova DS, Sologova SS, Tereshkina OI, Avakyan EI (2022) Update of Takotsubo cardiomyopathy: present experience and outlook for the future. Int J Cardiol Heart Vasc 39:100990 35281752 10.1016/j.ijcha.2022.100990 PMC8913320

[CR2] Bauer S, Hezinger L, Rexhepi F, Ramanathan S, Kufer TA (2023) NOD-like receptors-emerging links to obesity and associated morbidities. Int J Mol Sci 24:8595 37239938 10.3390/ijms24108595 PMC10218625

[CR3] Bertaggia Calderara D, Aliotta A, Zermatten MG, Kröll D, Stirnimann G, Alberio L (2020) Hyper-coagulability in obese patients accurately identified by combinations of global coagulation assay parameters. Thromb Res 187:91–102 31978812 10.1016/j.thromres.2020.01.012

[CR4] Brown SM, Larsen NK, Thankam FG, Agrawal DK (2021) Regulatory role of cardiomyocyte metabolism via AMPK activation in modulating atrial structural, contractile, and electrical properties following atrial fibrillation. Can J Physiol Pharmacol 99:36–41 33049144 10.1139/cjpp-2020-0313

[CR5] Butova X, Myachina T, Simonova R, Kochurova A, Mukhlynina E, Kopylova G, Shchepkin D, Khokhlova A (2023) The inter-chamber differences in the contractile function between left and right atrial cardiomyocytes in atrial fibrillation in rats. Front Cardiovasc Med 10:1203093 37608813 10.3389/fcvm.2023.1203093 PMC10440706

[CR6] Centuori SM, Bauman JE (2022) c-Met signaling as a therapeutic target in head and neck cancer. Cancer J 28:346–353 36165722 10.1097/PPO.0000000000000619

[CR7] Chitongo PB, Roberts LN, Yang L, Patel RK, Lyall R, Luxton R, Aylwin SJB, Arya R (2017) Visceral adiposity is an independent determinant of hypercoagulability as measured by thrombin generation in morbid obesity. TH Open 1:e146–e154 31249920 10.1055/s-0037-1608942 PMC6524850

[CR8] Cho S, Ying F, Sweeney G (2023) Sterile inflammation and the NLRP3 inflammasome in cardiometabolic disease. Biomed J 46:100624 37336361 10.1016/j.bj.2023.100624 PMC10539878

[CR9] Claycomb WC, Lanson NA Jr, Stallworth BS, Egeland DB, Delcarpio JB, Bahinski A, Izzo NJ Jr (1998) HL-1 cells: a cardiac muscle cell line that contracts and retains phenotypic characteristics of the adult cardiomyocyte. Proc Natl Acad Sci U S A 95:2979–2984 9501201 10.1073/pnas.95.6.2979 PMC19680

[CR10] Dai Y, Siemann DW (2012) Constitutively active c-Met kinase in PC-3 cells is autocrine-independent and can be blocked by the Met kinase inhibitor BMS-777607. BMC Cancer 12:198 22639908 10.1186/1471-2407-12-198 PMC3418572

[CR11] Dangwal S, Rauch BH, Gensch T, Dai L, Bretschneider E, Vogelaar CF, Schrör K, Rosenkranz AC (2011) High glucose enhances thrombin responses via protease-activated receptor-4 in human vascular smooth muscle cells. Arterioscler Thromb Vasc Biol 31:624–633 21164077 10.1161/ATVBAHA.110.219105

[CR12] Dobrev D, Heijman J, Hiram R, Li N, Nattel S (2023) Inflammatory signalling in atrial cardiomyocytes: a novel unifying principle in atrial fibrillation pathophysiology. Nat Rev Cardiol 20:145–167 36109633 10.1038/s41569-022-00759-w PMC9477170

[CR13] Dong X, Qin J, Ma J, Zeng Q, Zhang H, Zhang R, Liu C, Xu C, Zhang S, Huang S, Chen L (2019) BAFF inhibits autophagy promoting cell proliferation and survival by activating Ca(2+)-CaMKII-dependent Akt/mTOR signaling pathway in normal and neoplastic B-lymphoid cells. Cell Signal 53:68–79 30244168 10.1016/j.cellsig.2018.09.012 PMC6289808

[CR14] Fan X, Zhou J, Yan X, Bi X, Liang J, Lu S, Luo L, Zhou D, Yin Z (2021) Citrate activates autophagic death of prostate cancer cells via downregulation CaMKII/AKT/mTOR pathway. Life Sci 275:119355 33744326 10.1016/j.lfs.2021.119355

[CR15] Fender AC, Rauch BH, Geisler T, Schror K (2017) Protease-activated receptor PAR-4: an inducible switch between thrombosis and vascular inflammation? Thromb Haemost 117:2013–2025 29044290 10.1160/TH17-03-0219

[CR16] Fender AC, Wakili R, Dobrev D (2019) Straight to the heart: pleiotropic antiarrhythmic actions of oral anticoagulants. Pharmacol Res 145:104257 31054953 10.1016/j.phrs.2019.104257 PMC6987959

[CR17] Fender AC, Kleeschulte S, Stolte S, Leineweber K, Kamler M, Bode J, Li N, Dobrev D (2020) Thrombin receptor PAR4 drives canonical NLRP3 inflammasome signaling in the heart. Basic Res Cardiol 115:10 31912235 10.1007/s00395-019-0771-9 PMC7384378

[CR18] Fischer OM, Giordano S, Comoglio PM, Ullrich A (2004) Reactive oxygen species mediate Met receptor transactivation by G protein-coupled receptors and the epidermal growth factor receptor in human carcinoma cells. J Biol Chem 279:28970–28978 15123705 10.1074/jbc.M402508200

[CR19] Fleischer M, Szepanowski RD, Pesara V, Bihorac JS, Oehler B, Dobrev D, Kleinschnitz C, Fender AC (2024) Direct neuronal protection by the protease-activated receptor PAR4 antagonist ML354 after experimental stroke in mice. Br J Pharmacol 181(18):3364–3379. 10.1111/bph.16415 38760890 10.1111/bph.16415

[CR20] Gawałko M, Saljic A, Li N, Abu-Taha I, Jespersen T, Linz D, Nattel S, Heijman J, Fender A, Dobrev D (2023) Adiposity-associated atrial fibrillation: molecular determinants, mechanisms, and clinical significance. Cardiovasc Res 119:614–630 35689487 10.1093/cvr/cvac093 PMC10409902

[CR21] Gieseler F, Ungefroren H, Settmacher U, Hollenberg MD, Kaufmann R (2013) Proteinase-activated receptors (PARs) - focus on receptor-receptor-interactions and their physiological and pathophysiological impact. Cell Commun Signal 11:86 24215724 10.1186/1478-811X-11-86 PMC3842752

[CR22] Heijman J, Muna AP, Veleva T, Molina CE, Sutanto H, Tekook M, Wang Q, Abu-Taha IH, Gorka M, Künzel S, El-Armouche A, Reichenspurner H, Kamler M, Nikolaev V, Ravens U, Li N, Nattel S, Wehrens XHT, Dobrev D (2020) Atrial myocyte NLRP3/CaMKII nexus forms a substrate for postoperative atrial fibrillation. Circ Res 127:1036–1055 32762493 10.1161/CIRCRESAHA.120.316710 PMC7604886

[CR23] Hsu J, Hanna P, Van Wagoner DR, Barnard J, Serre D, Chung MK, Smith JD (2012) Whole genome expression differences in human left and right atria ascertained by RNA sequencing. Circ Cardiovasc Genet 5:327–335 22474228 10.1161/CIRCGENETICS.111.961631 PMC3380143

[CR24] Jin F, Lin Y, Yuan W, Wu S, Yang M, Ding S, Liu J, Chen Y (2024) Recent advances in c-Met-based dual inhibitors in the treatment of cancers. Eur J Med Chem 272:116477 38733884 10.1016/j.ejmech.2024.116477

[CR25] Kahr PC, Piccini I, Fabritz L, Greber B, Schöler H, Scheld HH, Hoffmeier A, Brown NA, Kirchhof P (2011) Systematic analysis of gene expression differences between left and right atria in different mouse strains and in human atrial tissue. PLoS One 6:e26389 22039477 10.1371/journal.pone.0026389 PMC3198471

[CR26] Kleeschulte S, Jerrentrup J, Gorski D, Schmitt J, Fender AC (2018) Evidence for functional PAR-4 thrombin receptor expression in cardiac fibroblasts and its regulation by high glucose: PAR-4 in cardiac fibroblasts. Int J Cardiol 252:163–166 29249425 10.1016/j.ijcard.2017.10.019

[CR27] Kleeschulte S, Fischinger V, Öhlke L, Bode J, Kamler M, Dobrev D, Grandoch M, Fender AC (2024) The thrombin receptor PAR4 supports visceral adipose tissue inflammation. Naunyn Schmiedebergs Arch Pharmacol. 10.1007/s00210-024-03097-5 10.1007/s00210-024-03097-5 PMC11422268 38652276

[CR28] Kudo M, Morimoto M, Moriguchi M, Izumi N, Takayama T, Yoshiji H, Hino K, Oikawa T, Chiba T, Motomura K, Kato J, Yasuchika K, Ido A, Sato T, Nakashima D, Ueshima K, Ikeda M, Okusaka T, Tamura K, Furuse J (2020) A randomized, double-blind, placebo-controlled, phase 3 study of tivantinib in Japanese patients with MET-high hepatocellular carcinoma. Cancer Sci 111:3759–3769 32716114 10.1111/cas.14582 PMC7541009

[CR29] Leo C, Sala V, Morello M, Chiribiri A, Riess I, Mancardi D, Schiaffino S, Ponzetto C, Crepaldi T (2011) Activated Met signalling in the developing mouse heart leads to cardiac disease. PLoS One 6:e14675 21347410 10.1371/journal.pone.0014675 PMC3036588

[CR30] Li H, Wang H, Wang T, Jin C, Lu M, Liu B (2023) Different phenotype of left atrial function impairment in patients with hypertrophic cardiomyopathy and hypertension: comparison of healthy controls. Front Cardiovasc Med 10:1027665 37234371 10.3389/fcvm.2023.1027665 PMC10206117

[CR31] Lin H, Liu AP, Smith TH, Trejo J (2013) Cofactoring and dimerization of proteinase-activated receptors. Pharmacol Rev 65:1198–1213 24064459 10.1124/pr.111.004747 PMC3799237

[CR32] Lu MK, Huo YN, Tai BY, Lin CY, Yang HY, Tsai CS (2024) Ziprasidone triggers inflammasome signaling via PI3K-Akt-mTOR pathway to promote atrial fibrillation. Biomed Pharmacother 175:116649 38692059 10.1016/j.biopha.2024.116649

[CR33] Malandish A, Gulati M (2023) The impacts of exercise interventions on inflammaging markers in overweight/obesity patients with heart failure: a systematic review and meta-analysis of randomized controlled trials. Int J Cardiol Heart Vasc 47:101234 37416483 10.1016/j.ijcha.2023.101234 PMC10320319

[CR34] Mann C, van Alst C, Gorressen S, Nega R, Dobrev D, Grandoch M, Fender AC (2024) Ischemia does not provoke the full immune training repertoire in human cardiac fibroblasts. Naunyn Schmiedebergs Arch Pharmacol. 10.1007/s00210-024-03107-6 10.1007/s00210-024-03107-6 PMC11422419 38652279

[CR35] Merali S, Wang Z, Frost C, Meadows-Shropshire S, Hawthorne D, Yang J, Seiffert D (2023) First-in-human study to assess the safety, pharmacokinetics, and pharmacodynamics of BMS-986141, a novel, reversible, small-molecule, PAR4 agonist in non-Japanese and Japanese healthy participants. Platelets 34:2222846 37394920 10.1080/09537104.2023.2222846

[CR36] Mihara K, Ramachandran R, Saifeddine M, Hansen KK, Renaux B, Polley D, Gibson S, Vanderboor C, Hollenberg MD (2016) Thrombin-mediated direct activation of proteinase-activated receptor-2: another target for thrombin signaling. Mol Pharmacol 89:606–614 26957205 10.1124/mol.115.102723

[CR37] Nash J, Meah MN, Whittington B, Debono S, Raftis J, Miller MR, Sorbie A, Mills NL, Nespoux J, Bruce L, Duffin R, Dhaun N, Brittan M, Chao L, Merali S, Kim M, Wang Z, Zhang Y, Jin S, Wang B, Kozinn M, Newby DE (2024) PAR4 antagonism in patients with coronary artery disease receiving antiplatelet therapies. Arterioscler Thromb Vasc Biol 44:987–996 38357820 10.1161/ATVBAHA.123.320448

[CR38] Ozcan C, Dixit G, Li Z (2021) Activation of AMP-activated protein kinases prevents atrial fibrillation. J Cardiovasc Transl Res 14:492–502 32844365 10.1007/s12265-020-10069-6

[CR39] Pavic G, Grandoch M, Dangwal S, Jobi K, Rauch BH, Doller A, Oberhuber A, Akhyari P, Schrör K, Fischer JW, Fender AC (2014) Thrombin receptor protease-activated receptor 4 is a key regulator of exaggerated intimal thickening in diabetes mellitus. Circulation 130:1700–1711 25239438 10.1161/CIRCULATIONAHA.113.007590

[CR40] Pinet C, Algalarrondo V, Sablayrolles S, Le Grand B, Pignier C, Cussac D, Perez M, Hatem SN, Coulombe A (2008) Protease-activated receptor-1 mediates thrombin-induced persistent sodium current in human cardiomyocytes. Mol Pharmacol 73:1622–1631 18326052 10.1124/mol.107.043182

[CR41] Ruf L, Bukowska A, Gardemann A, Goette A (2023) Coagulation factor Xa has no effects on the expression of PAR1, PAR2, and PAR4 and no proinflammatory effects on HL-1 cells. Cells 12:2849 38132169 10.3390/cells12242849 PMC10741780

[CR42] Rusciano D, Lorenzoni P, Burger MM (1996) Constitutive activation of c-Met in liver metastatic B16 melanoma cells depends on both substrate adhesion and cell density and is regulated by a cytosolic tyrosine phosphatase activity. J Biol Chem 271:20763–20769 8702829 10.1074/jbc.271.34.20763

[CR43] Schrottmaier WC, Assinger A (2024) The concept of thromboinflammation. Hamostaseologie 44:21–30 38417802 10.1055/a-2178-6491 PMC12997454

[CR44] Scott L Jr, Fender AC, Saljic A, Li L, Chen X, Wang X, Linz D, Lang J, Hohl M, Twomey D, Pham TT, Diaz-Lankenau R, Chelu MG, Kamler M, Entman ML, Taffet GE, Sanders P, Dobrev D, Li N (2021) NLRP3 inflammasome is a key driver of obesity-induced atrial arrhythmias. Cardiovasc Res 117:1746–1759 33523143 10.1093/cvr/cvab024 PMC8208743

[CR45] Sidhu TS, French SL, Hamilton JR (2014) Differential signaling by protease-activated receptors: implications for therapeutic targeting. Int J Mol Sci 15:6169–6183 24733067 10.3390/ijms15046169 PMC4013622

[CR46] Stark K, Massberg S (2021) Interplay between inflammation and thrombosis in cardiovascular pathology. Nat Rev Cardiol 18:666–682 33958774 10.1038/s41569-021-00552-1 PMC8100938

[CR47] Su KN, Ma Y, Cacheux M, Ilkan Z, Raad N, Muller GK, Wu X, Guerrera N, Thorn SL, Sinusas AJ, Foretz M, Viollet B, Akar JG, Akar FG, Young LH (2022) Atrial AMP-activated protein kinase is critical for prevention of dysregulation of electrical excitability and atrial fibrillation. JCI Insight 7:e141213 35451373 10.1172/jci.insight.141213 PMC9089788

[CR48] Vahdat S (2022) A review of pathophysiological mechanism, diagnosis, and treatment of thrombosis risk associated with COVID-19 infection. Int J Cardiol Heart Vasc 41:101068 35677840 10.1016/j.ijcha.2022.101068 PMC9163146

[CR49] Voigt N, Zhou XB, Dobrev D (2013) Isolation of human atrial myocytes for simultaneous measurements of Ca2+ transients and membrane currents. J Vis Exp 3:e50235 10.3791/50235 PMC3731177 23852392

[CR50] Wong LY, Glatz JFC, Wang S, Geraets IME, Vanherle S, Wijngaard AVD, Brunner H, Luiken J, Nabben M (2021) Comparison of human and rodent cell models to study myocardial lipid-induced insulin resistance. Prostaglandins Leukot Essent Fatty Acids 167:102267 33751940 10.1016/j.plefa.2021.102267

[CR51] Wu J, Xu H, Li H, Ma L, Chen J, Yuan F, Sheng L, Liu C, Chen W, Li X (2023) Effect of food on the pharmacokinetics and safety of a novel c-Met inhibitor SCC244: a randomized phase I study in healthy subjects. Drug Des Devel Ther 17:761–769 36925997 10.2147/DDDT.S388846 PMC10013581

[CR52] Yao C, Veleva T, Scott L Jr, Cao S, Li L, Chen G, Jeyabal P, Pan X, Alsina KM, Abu-Taha ID, Ghezelbash S, Reynolds CL, Shen YH, LeMaire SA, Schmitz W, Müller FU, El-Armouche A, Tony Eissa N, Beeton C, Nattel S, Wehrens XHT, Dobrev D, Li N (2018) Enhanced cardiomyocyte NLRP3 inflammasome signaling promotes atrial fibrillation. Circulation 138:2227–2242 29802206 10.1161/CIRCULATIONAHA.118.035202 PMC6252285

