# Correction: Thrombin receptor PAR4 cross-activates the tyrosine kinase c-met in atrial cardiomyocytes

**DOI:** 10.1007/s00210-024-03703-6

**Published:** 2024-12-10

**Authors:** Claudia Mittendorff, Issam Abu-Taha, Lena Kassler, Tobias Hustedt, Stephanie Wolf, Johannes G. Bode, Markus Kamler, Dobromir Dobrev, Anke C. Fender

**Affiliations:** 1 Institute of Pharmacology, West German Heart and Vascular Center, University Duisburg-Essen, Duisburg, Germany; 2 Department of Gastroenterology, Hepatology and Infectious disease, Faculty of Medicine & Düsseldorf University Hospital, Heinrich-Heine-University, Düsseldorf, Germany; 3 Department of Thoracic and Cardiovascular Surgery, University Hospital Essen, Essen, Germany; 4 Department of Medicine and Research Center, Montreal Heart Institute and Université de Montréal, Montréal, Canada; 5 Department of Integrative Physiology, Baylor College of Medicine, Houston, TX USA

**Correction: Naunyn-Schmiedeberg's Archives of Pharmacology**

10.1007/s00210-024-03436-6

The article “Thrombin receptor PAR4 cross‑activates the tyrosine kinase c‑met in atrial cardiomyocytes”, written by Claudia Mittendorff, Issam Abu‑Taha, Lena Kassler, Tobias Hustedt, Stephanie Wolf, Johannes G. Bode, Markus Kamler, Dobromir Dobrev and Anke C. Fender, was originally published electronically on the publisher’s internet portal on 16 September 2024 without open access. With the author(s)’ decision to opt for Open Choice the copyright of the article changed on 28 November 2024 to © The Author(s) 2024 and the article is forthwith distributed under a Creative Commons Attribution 4.0 International License, which permits use, sharing, adaptation, distribution and reproduction in any medium or format, as long as you give appropriate credit to the original author(s) and the source, provide a link to the Creative Commons licence, and indicate if changes were made. The images or other third party material in this article are included in the article’s Creative Commons licence, unless indicated otherwise in a credit line to the material. If material is not included in the article’s Creative Commons licence and your intended use is not permitted by statutory regulation or exceeds the permitted use, you will need to obtain permission directly from the copyright holder. To view a copy of this licence, visit http://creativecommons.org/licenses/by/4.0. Open Access funding enabled and organized by Projekt DEAL.

The original article was corrected.

