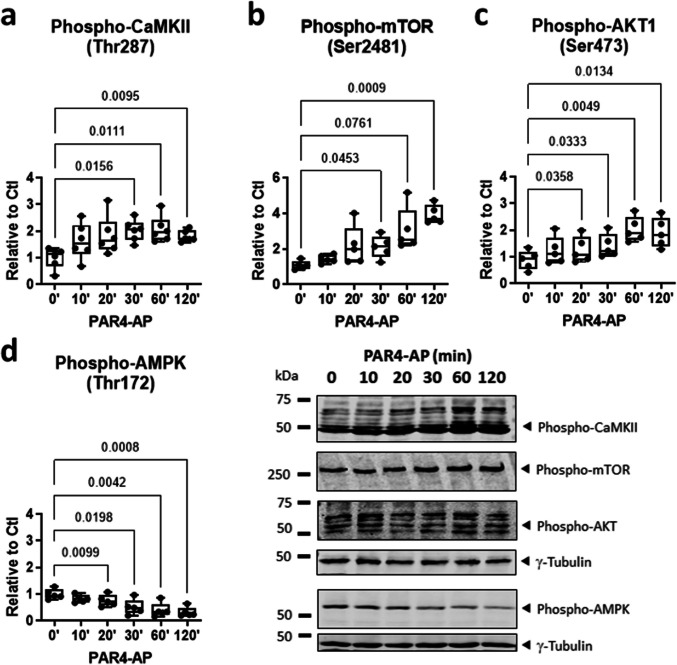# Correction: Thrombin receptor PAR4 cross-activates the tyrosine kinase c-met in atrial cardiomyocytes

**DOI:** 10.1007/s00210-025-03953-y

**Published:** 2025-09-09

**Authors:** Claudia Mittendorff, Issam Abu-Taha, Lena Kassler, Tobias Hustedt, Stephanie Wolf, Johannes G. Bode, Markus Kamler, Dobromir Dobrev, Anke C. Fender

**Affiliations:** 1 Institute of Pharmacology, West German Heart and Vascular Center, University Duisburg-Essen, Duisburg, Germany; 2 Department of Gastroenterology, Hepatology and Infectious Disease, Faculty of Medicine & Düsseldorf University Hospital, Heinrich-Heine-University, Düsseldorf, Germany; 3 Department of Thoracic and Cardiovascular Surgery, University Hospital Essen, Essen, Germany; 4 Department of Medicine and Research Center, Montreal Heart Institute and Université de Montréal, Montréal, Canada; 5 Department of Integrative Physiology, Baylor College of Medicine, Houston, TX USA

**Correction to** **:** **Naunyn Schmiedebergs Arch Pharmacol 2024 Sep 16**

10.1007/s00210-024-03436-6

The authors regret that Fig. 3a, depicting phosphorylated CaMKII, contains an incorrect data set. During revision, the data set for Fig. 3b (phosphorylated mTOR) was inadvertently copied over. We wish to provide a corrigendum of Fig. 3 to ensure scientific integrity and fulfil the high standard of *Naunyn-Schmiedebergs Archives of Pharmacology*. Only the graphical representation of Fig. 3a is changed; the data interpretation and study conclusions are unaffected. We apologise for this error and provide the correct figure below: