# Supplementary material for: Thrombin receptor PAR4 cross-activates the tyrosine kinase c-met in atrial cardiomyocytes
Source: Naunyn Schmiedebergs Arch Pharmacol. 2024 Sep 16;398(3):2783–96. doi: 10.1007/s00210-024-03436-6 (PMC11920351; doi:10.1007/s00210-024-03436-6)
Supplement: Supplementary file 1 — Supplementary file1 (PDF 722 KB) [file 210_2024_3436_MOESM1_ESM.pdf]

Thrombin receptor PAR4 cross-activates the receptor tyrosine kinase c-met in atrial cardiomyocytes

Claudia Mittendorff,[1#] Issam Abu-Taha,[1#] Lena Kassler,[1#] Tobias Hustedt,[2#] Stephanie Wolf,[2#] Johannes G. Bode,[2#] Markus Kamler,[3#] Dobromir Dobrev[1,4,5#], Anke C. Fender[1#]

[1]Institute of Pharmacology, West German Heart and Vascular Center, University Duisburg-Essen, Duisburg, Germany
[2]Department of Gastroenterology, Hepatology and Infectious disease, Faculty of Medicine & Düsseldorf University Hospital, Heinrich-Heine-University, Düsseldorf, Germany
[3]Department of Thoracic and Cardiovascular Surgery, University Hospital Essen, Germany
[4]Department of Medicine and Research Center, Montreal Heart Institute and Université de Montréal, Montréal, Canada
[5]Department of Integrative Physiology, Baylor College of Medicine, Houston, TX, USA
[#]This author takes responsibility for all aspects of the reliability and freedom from bias of the data presented and their discussed interpretation

Data supplement 1

Total protein immunoblots

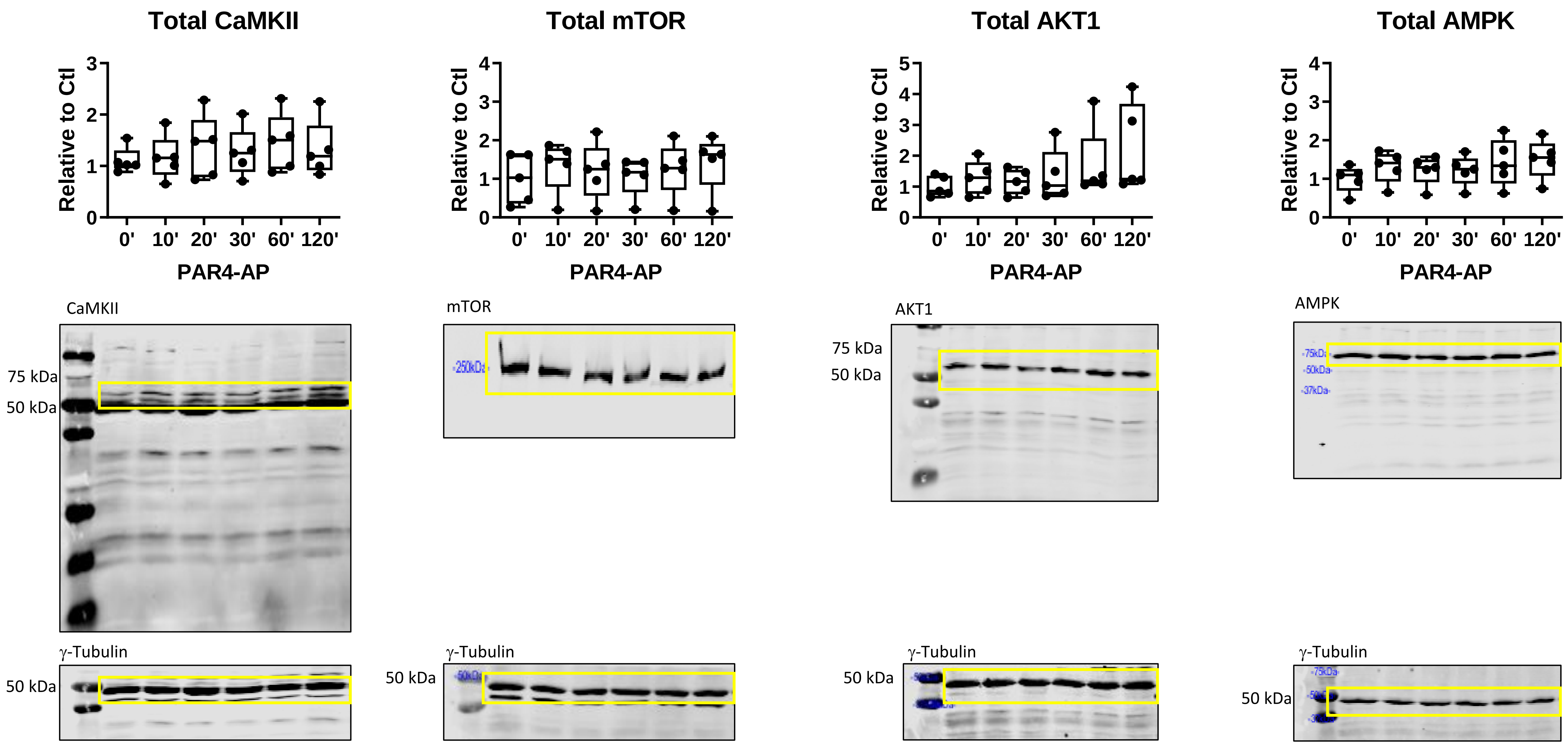

Suppl. Fig. S1 Total protein expression of CaMKII, mTOR, AKT and AMPK, all normalised to γ-tubulin, in HL-1 cells stimulated with PAR4-activating peptide (AP) for the indicated times, all n=5. These images correspond to the phospho-proteins depicted in main Fig. 3.

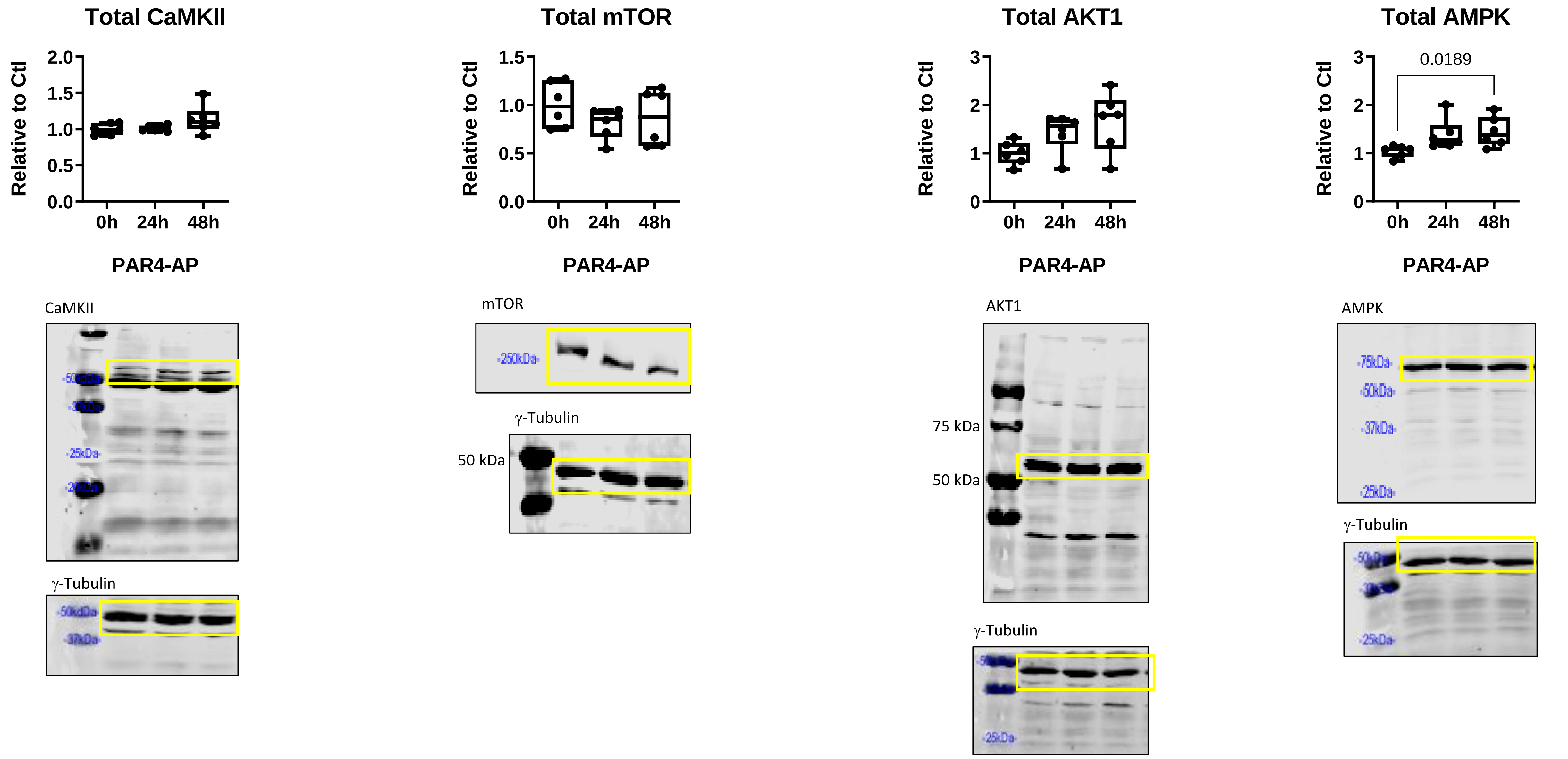

Suppl. Fig. S2 Total protein expression of CaMKII, mTOR, AKT and AMPK, all normalised to γ-tubulin, in HL-1 cells stimulated with PAR4-activating peptide (AP) for the indicated times, all n=5. These images correspond to the phospho-proteins depicted in main Fig. 4.

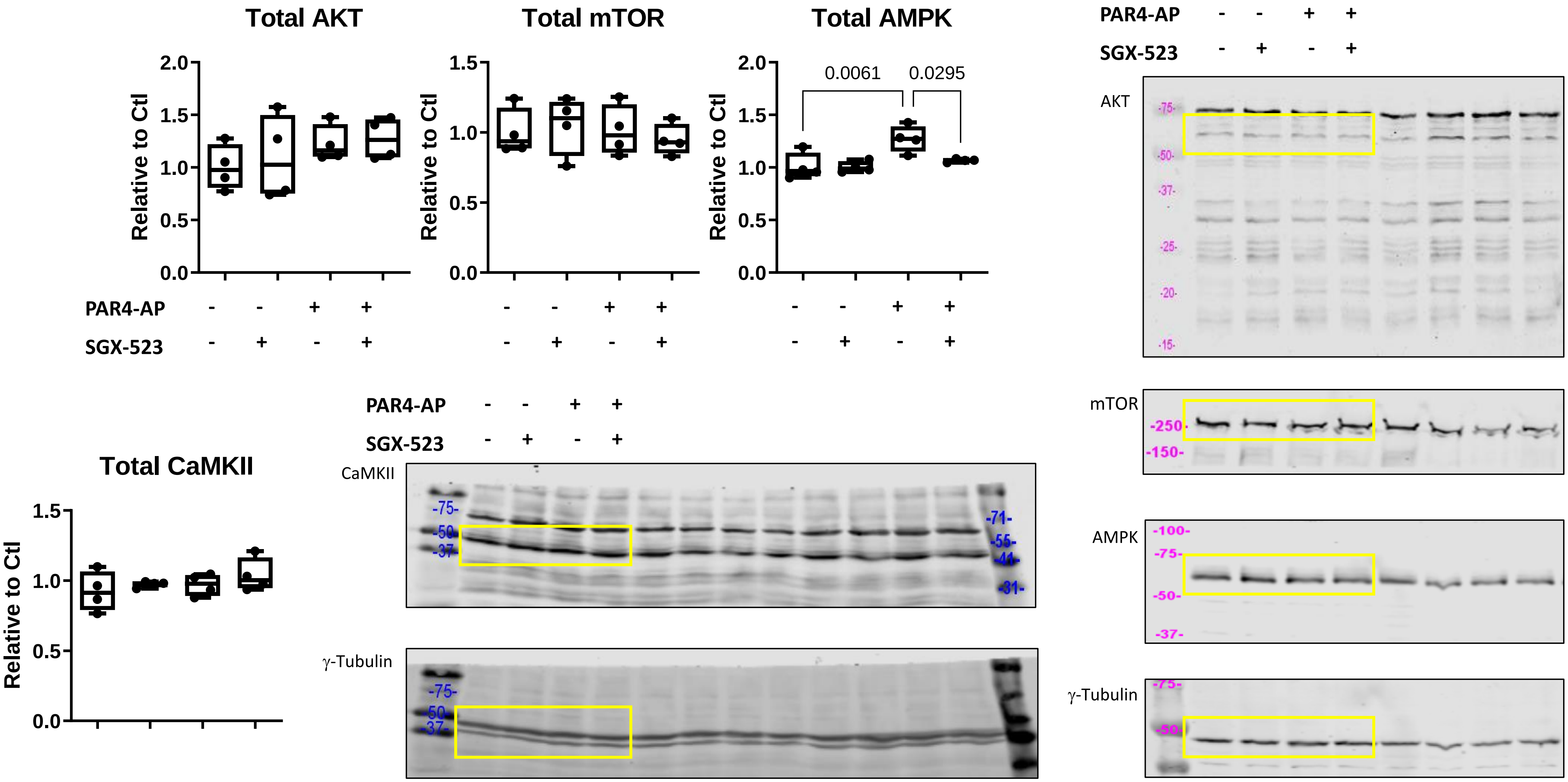

Suppl. Fig. S3 Total protein expression of CaMKII, AKT, mTOR, and AMPK, all normalised to γ-tubulin, in HL-1 cells stimulated with PAR4-activating peptide (AP) for the indicated times, all n=4. These images correspond to the phospho-proteins depicted in main Fig. 7

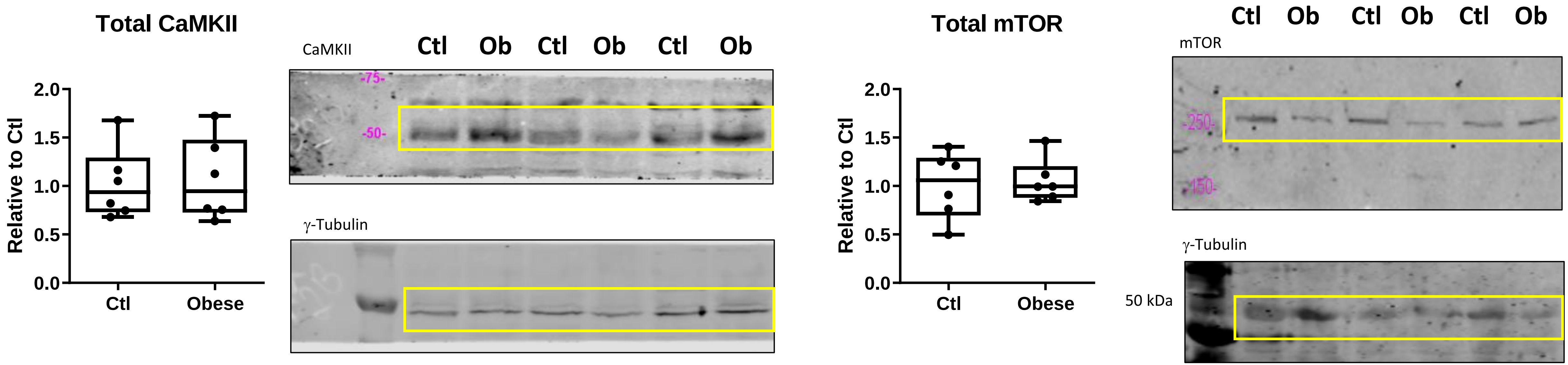

Suppl. Fig. S4 Total CAMKII and mTOR protein expression, all normalised to γ-tubulin, in right atrial appendage lysates from obese versus non-obese control (Ctl) patients undergoing cardiac surgery, all n=6. These images correspond to the phospho-proteins depicted in main Fig. 8.

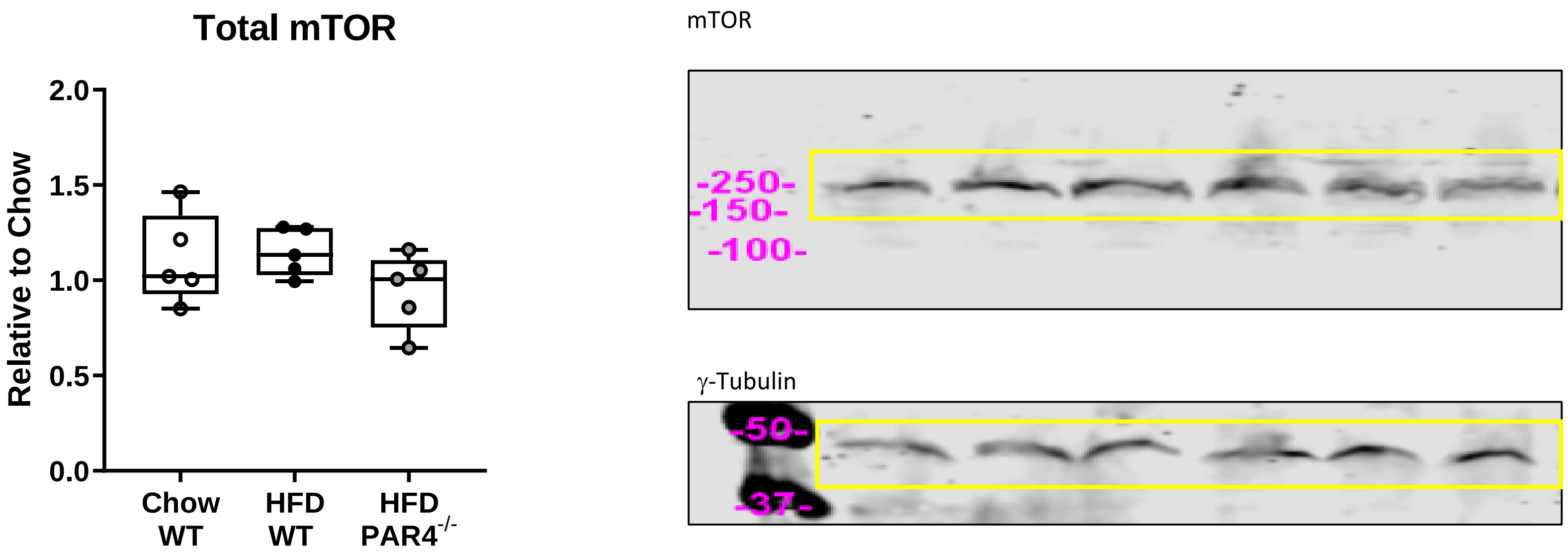

Suppl. Fig. S5 Total mTOR protein expression, normalised to γ-tubulin, in atrial lysates of wildtype (WT) or PAR4$^{-/-}$ mice fed chow or a high fat diet (HFD) for 8 weeks, all n=5, corresponding to phospho-mTOR depicted in main Fig. 9.
