# Supplementary material for: Thrombin receptor PAR4 cross-activates the tyrosine kinase c-met in atrial cardiomyocytes
Source: Naunyn Schmiedebergs Arch Pharmacol. 2024 Sep 16;398(3):2783–96. doi: 10.1007/s00210-024-03436-6 (PMC11920351; doi:10.1007/s00210-024-03436-6)

Thrombin receptor PAR4 cross-activates the receptor tyrosine kinase c-met in atrial cardiomyocytes

Claudia Mittendorff,[1#] Issam Abu-Taha,[1#] Lena Kassler,[1#] Tobias Hustedt,[2#] Stephanie Wolf,[2#] Johannes G. Bode,[2#] Markus Kamler,[3#] Dobromir Dobrev[1,4,5#], Anke C. Fender[1#]

[1]Institute of Pharmacology, West German Heart and Vascular Center, University Duisburg-Essen, Duisburg, Germany
[2]Department of Gastroenterology, Hepatology and Infectious disease, Faculty of Medicine & Düsseldorf University Hospital, Heinrich-Heine-University, Düsseldorf, Germany
[3]Department of Thoracic and Cardiovascular Surgery, University Hospital Essen, Germany
[4]Department of Medicine and Research Center, Montreal Heart Institute and Université de Montréal, Montréal, Canada
[5]Department of Integrative Physiology, Baylor College of Medicine, Houston, TX, USA
[#]This author takes responsibility for all aspects of the reliability and freedom from bias of the data presented and their discussed interpretation

Data supplement 2

Uncut immunoblots: yellow boxes indicate sections shown in main figure

Figure 1

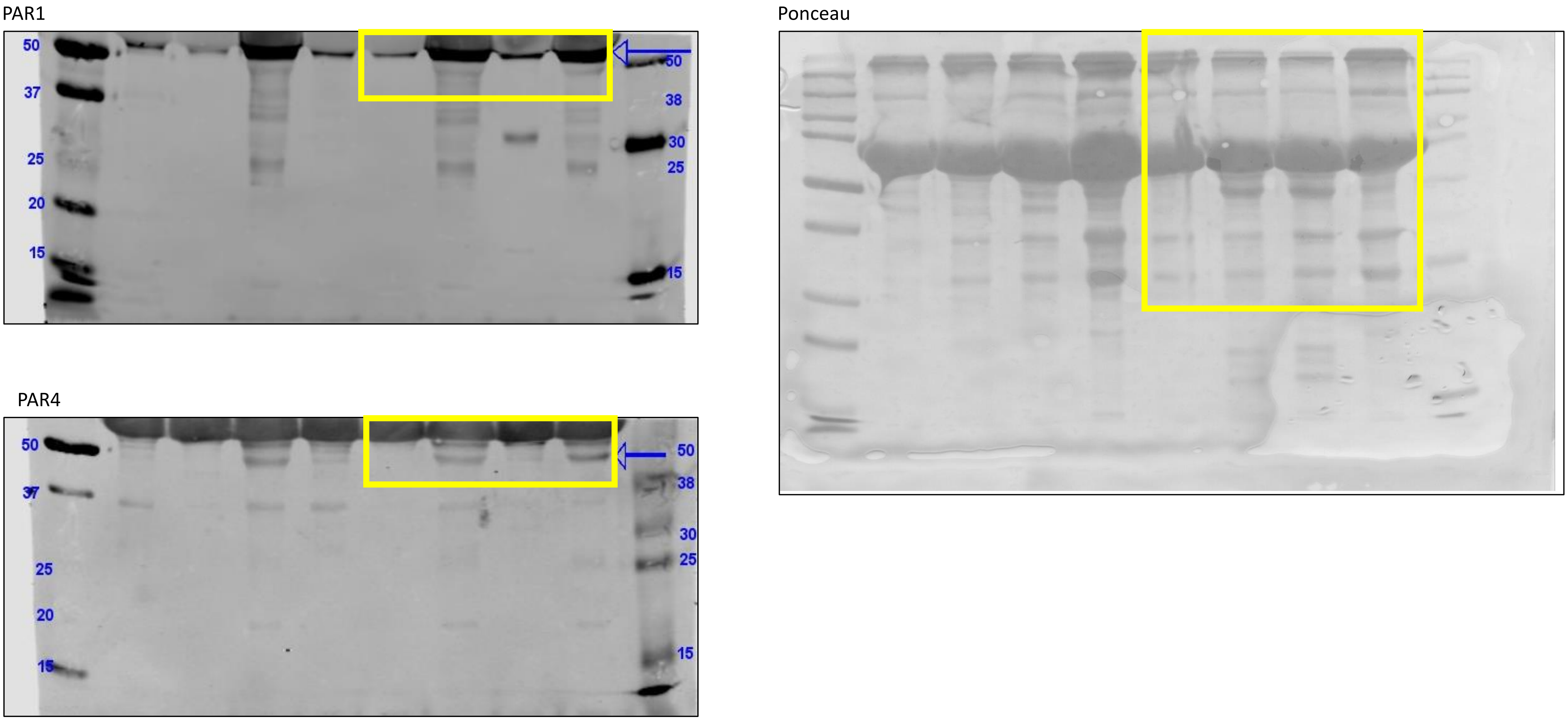
PAR1
50
37
25
20
15
50
38
30
25
15
PAR4
50
37
25
20
15
50
38
30
25
15
Ponceau

Figure 2

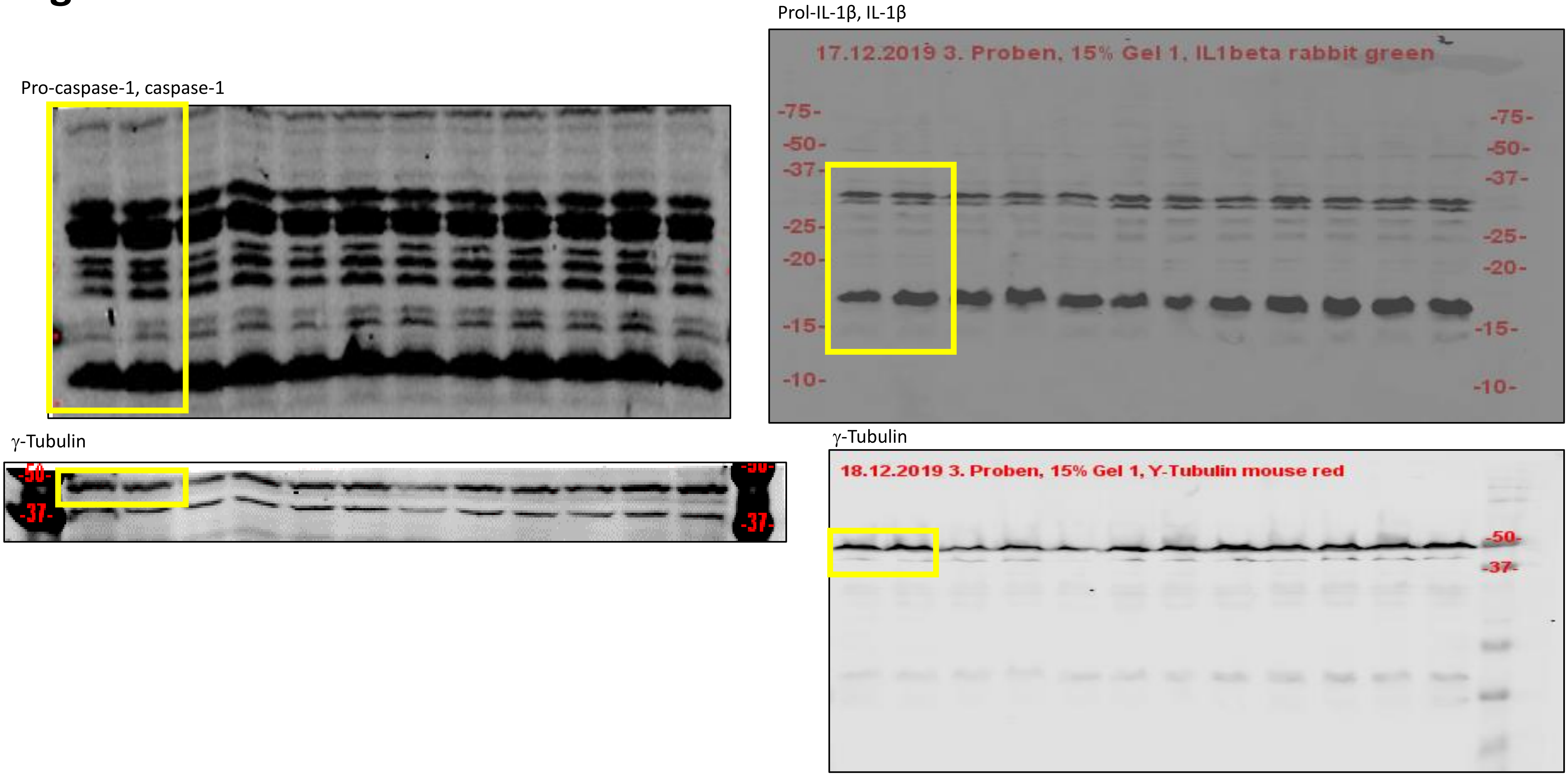

Pro-caspase-1, caspase-1
Prol-IL-1β, IL-1β
17.12.2019 3. Proben, 15% Gel 1, IL1beta rabbit green
-75-
-50-
-37-
-25-
-20-
-15-
-10-
γ-Tubulin
-50-
-37-
γ-Tubulin
18.12.2019 3. Proben, 15% Gel 1, Y-Tubulin mouse red
-50-
-37-

Figure 3

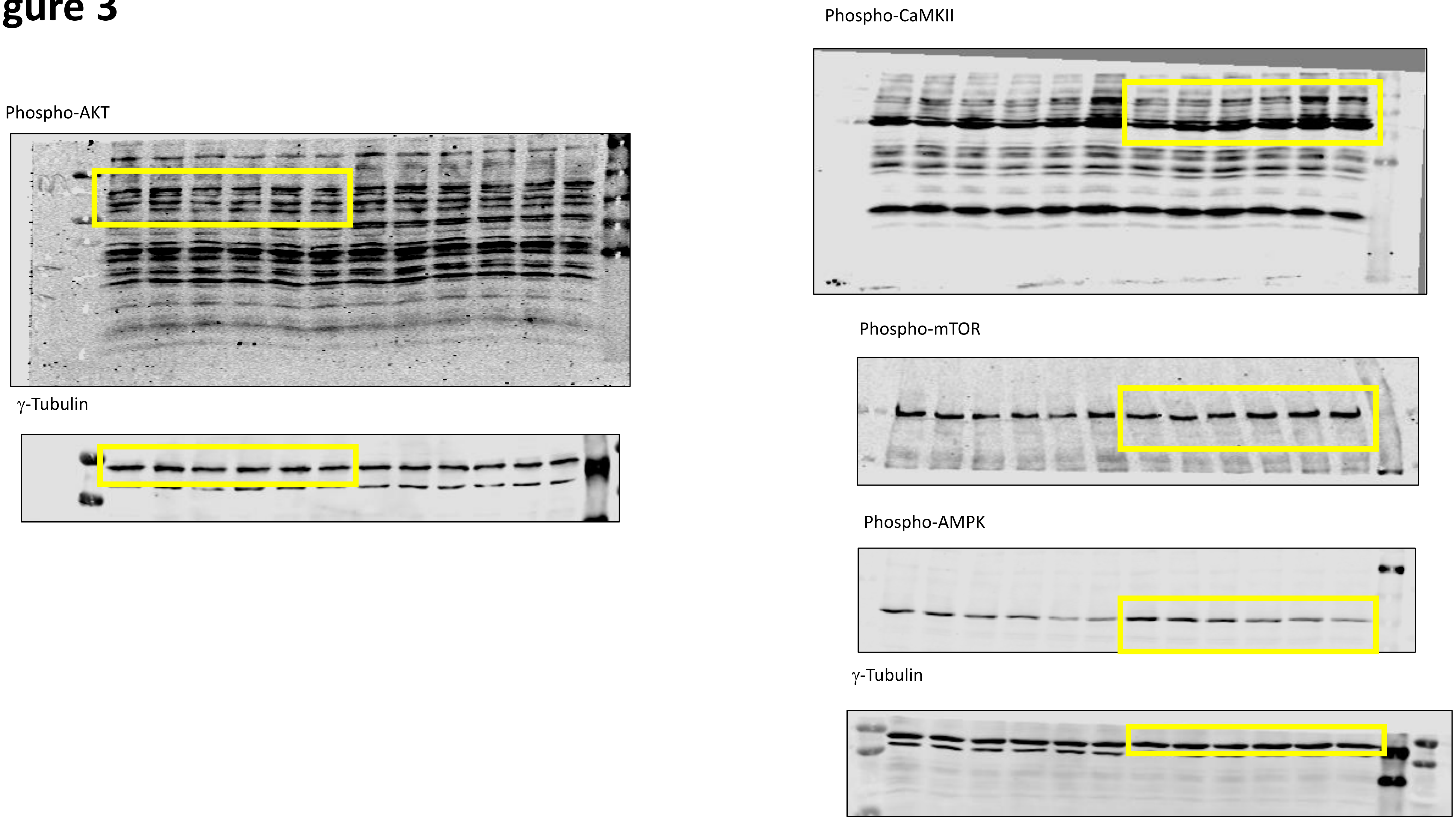
Phospho-AKT
γ-Tubulin
Phospho-CaMKII
Phospho-mTOR
Phospho-AMPK
γ-Tubulin

Figure 4

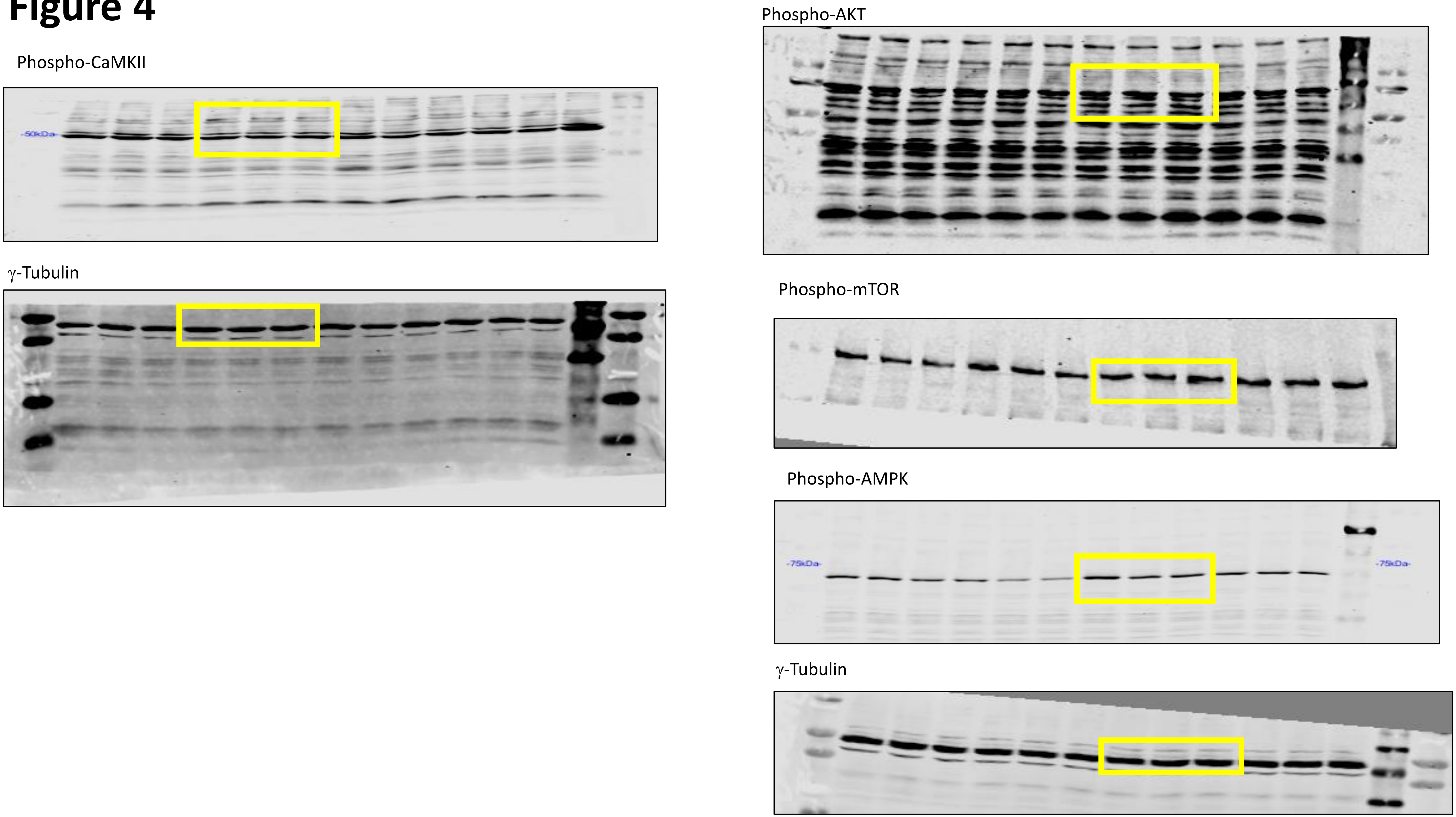
Phospho-CaMKII
-50kDa-
γ-Tubulin
Phospho-AKT
Phospho-mTOR
Phospho-AMPK
-75kDa-
-75kDa-
γ-Tubulin

Figure 5

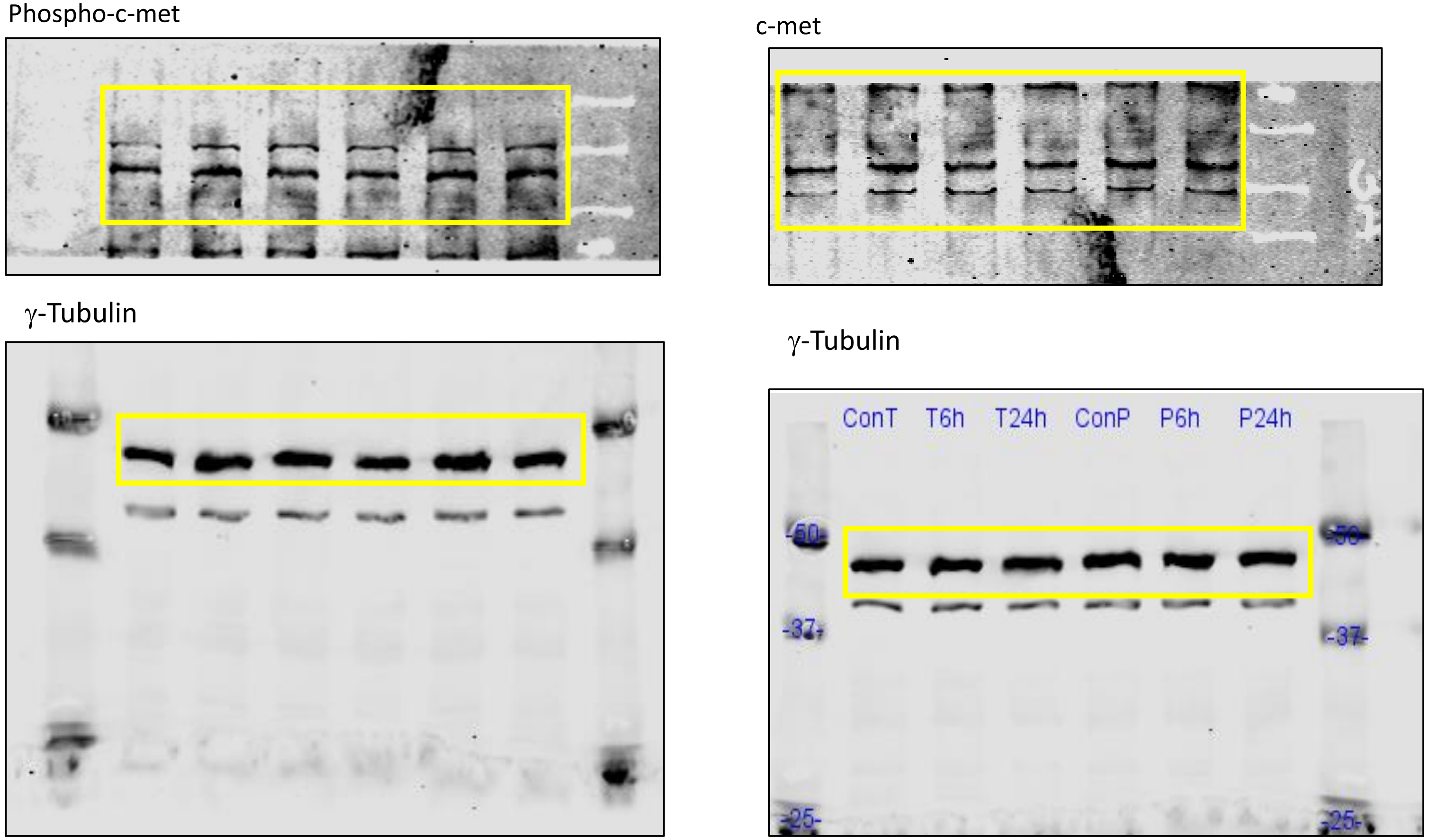
Phospho-c-met
c-met
γ-Tubulin
γ-Tubulin
ConT
T6h
T24h
ConP
P6h
P24h
50
37
25
50
37
25

Figure 6

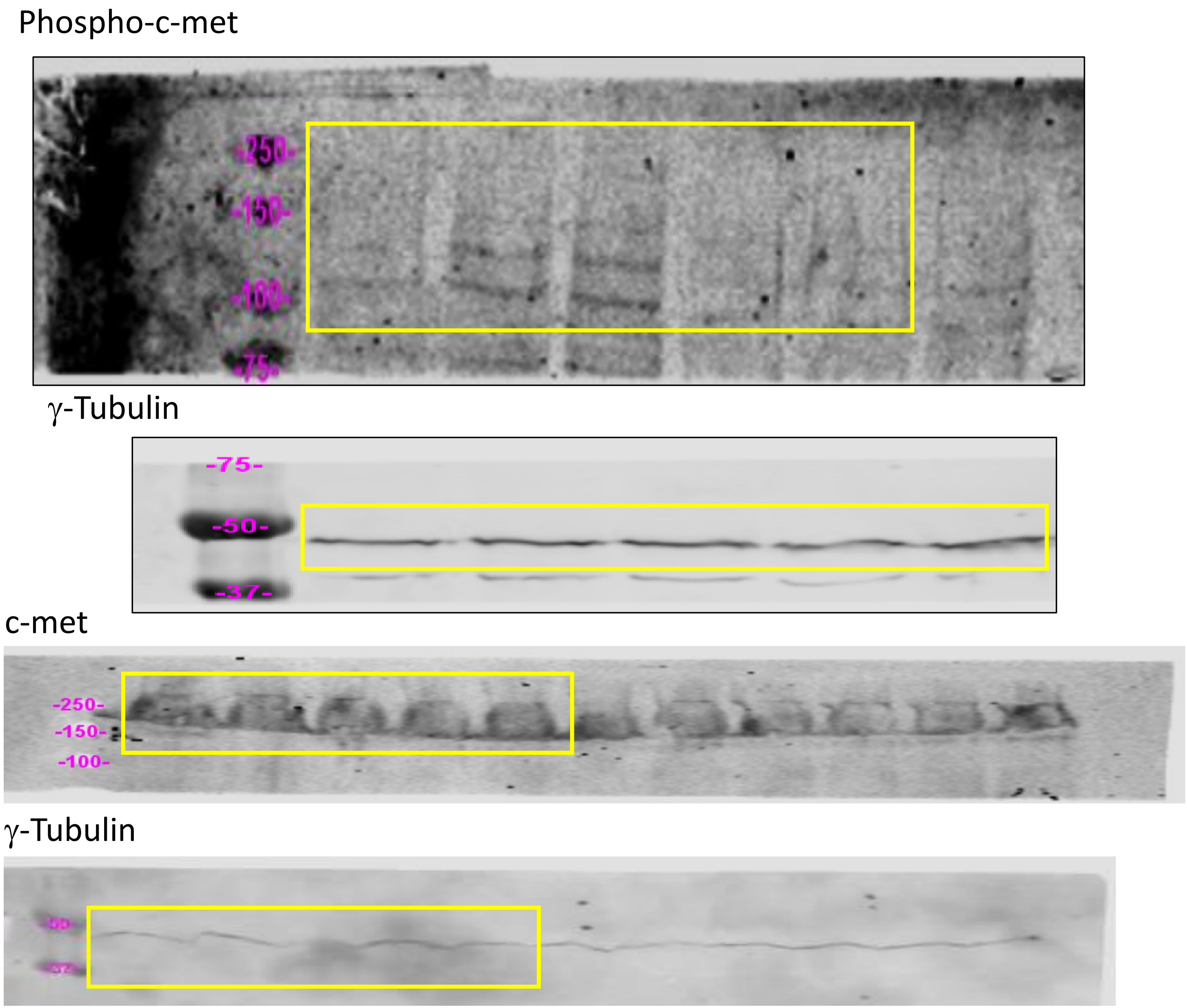
Phospho-c-met
250
150
100
75
γ-Tubulin
-75-
-50-
-37-
c-met
-250-
-150-
-100-
γ-Tubulin

Figure 7 (1 of 2)

p-CaMKII

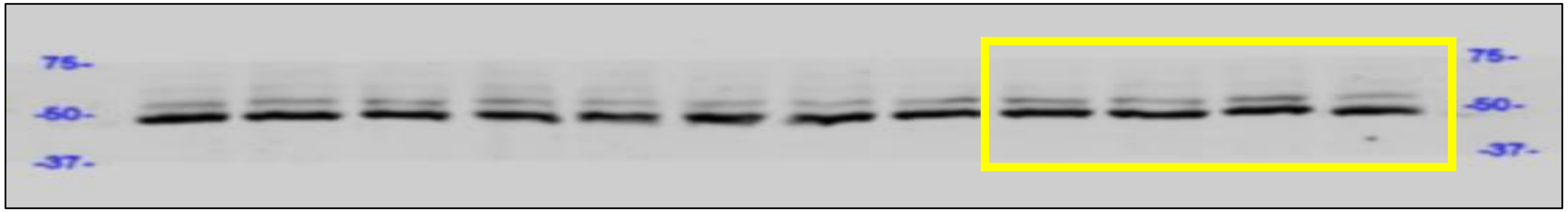

γ-Tubulin

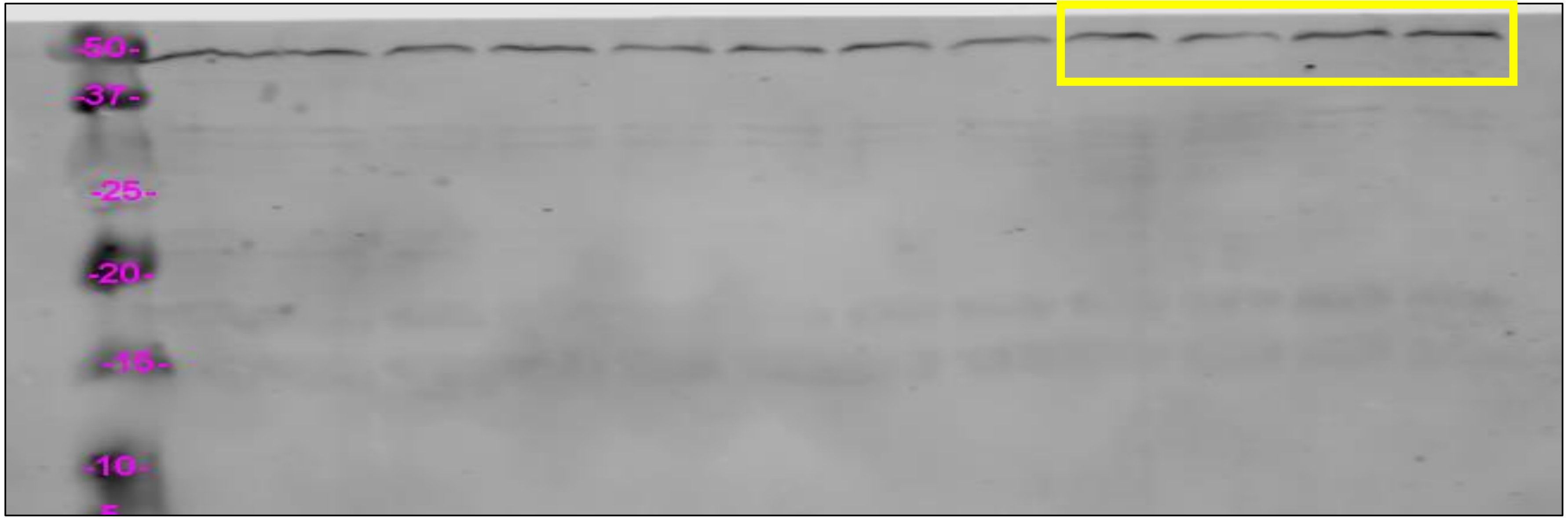

Figure 7 (2 of 2)

p-AMPK

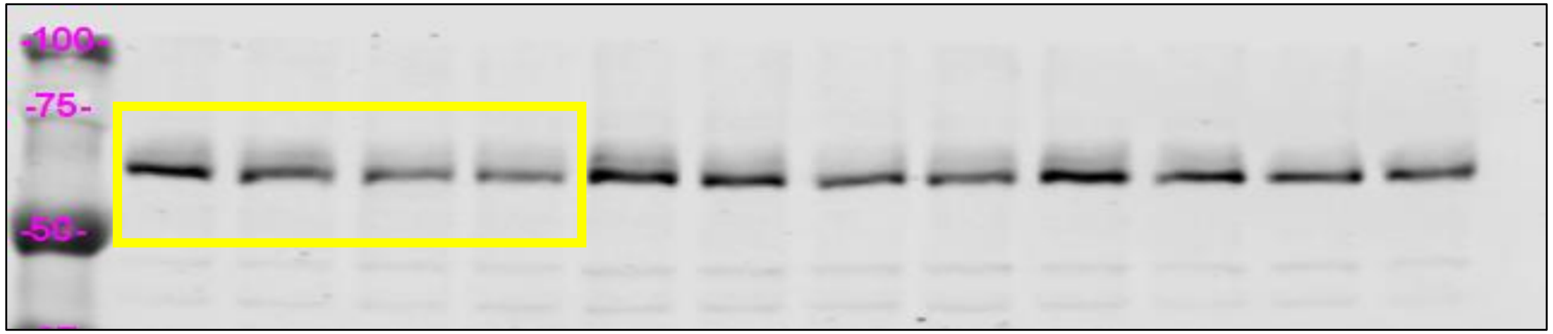

p-mTOR

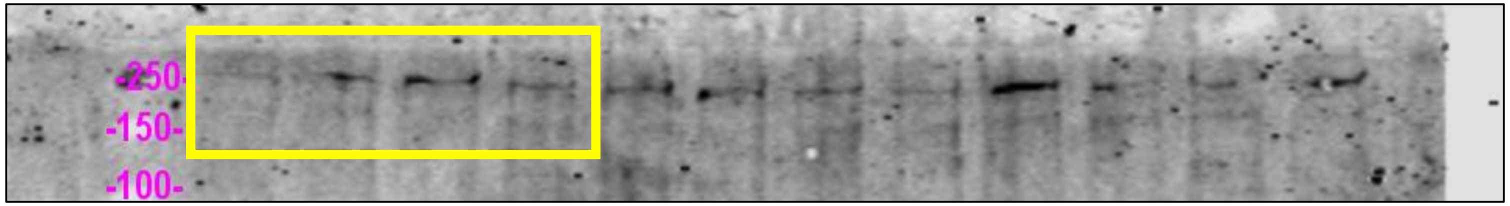

γ-Tubulin

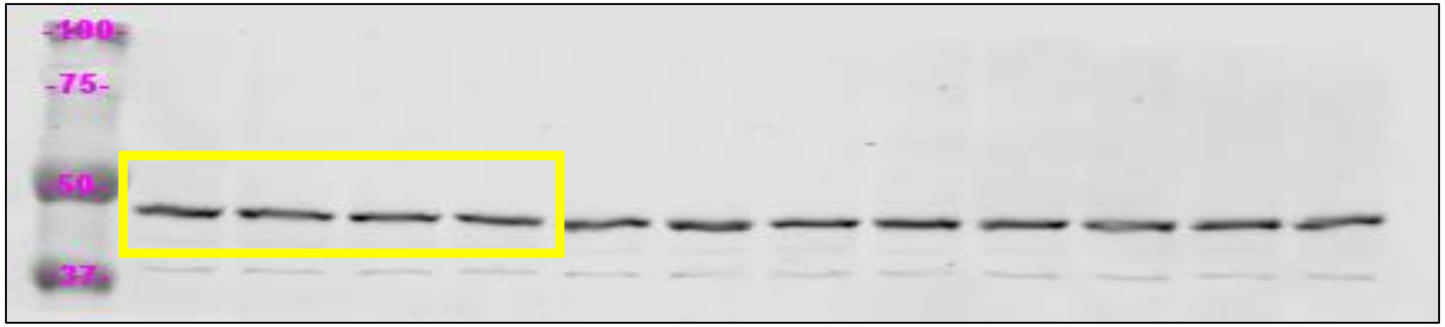

Figure 8

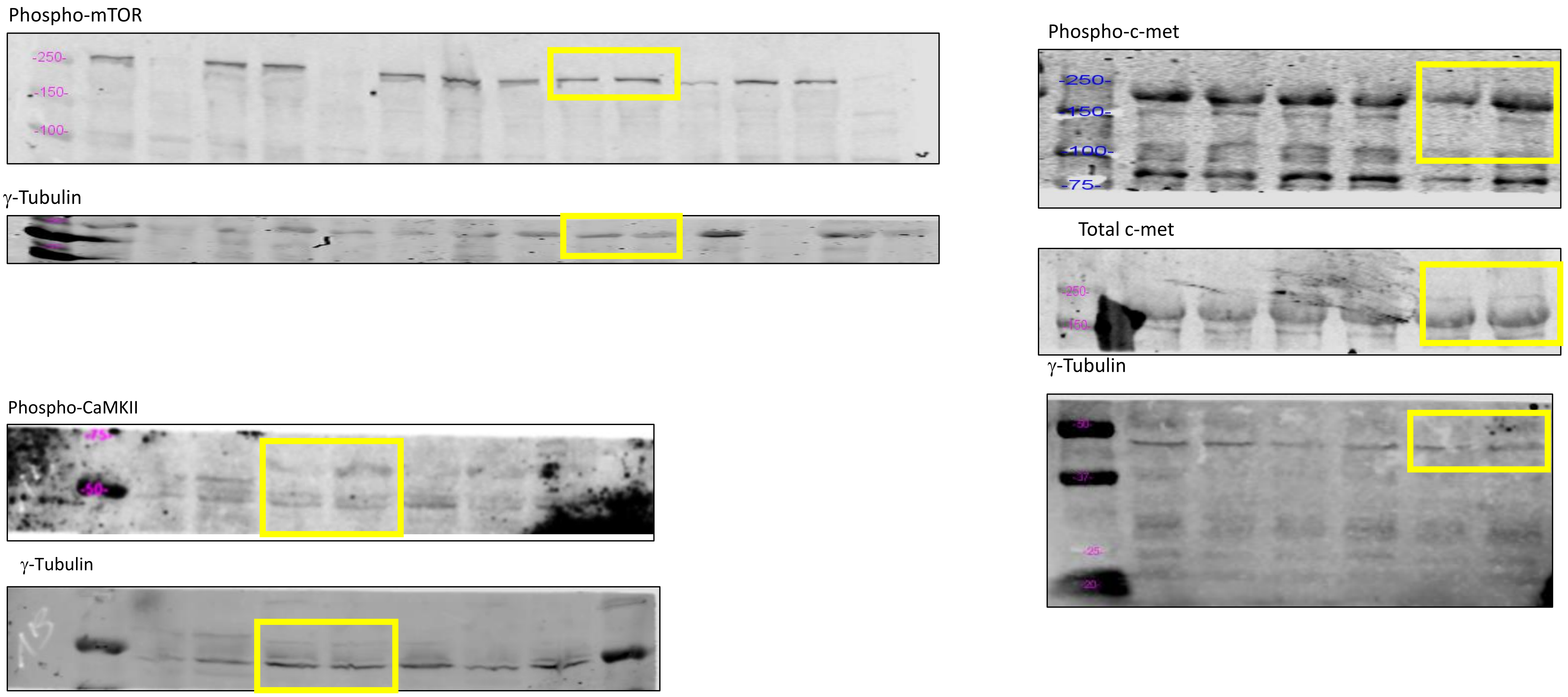

Phospho-mTOR
-250-
-150-
-100-
γ-Tubulin
Phospho-CaMKII
-50-
γ-Tubulin
Phospho-c-met
-250-
-150-
-100-
-75-
Total c-met
-250-
-150-
γ-Tubulin
-50-
-37-
-25-
-20-

Figure 9

Phospho-mTOR

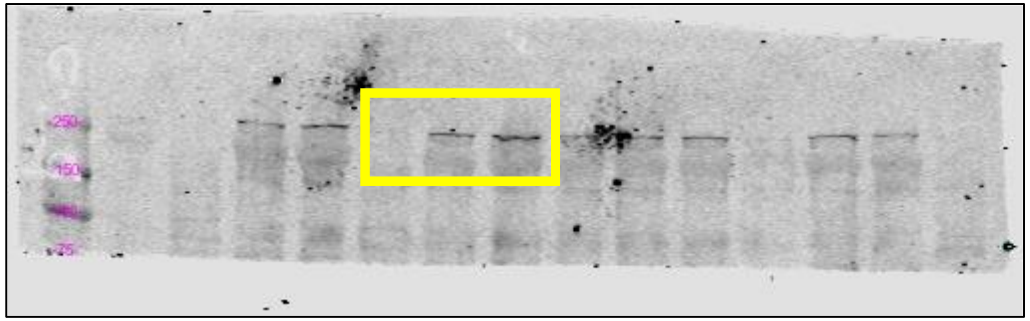

γ-Tubulin

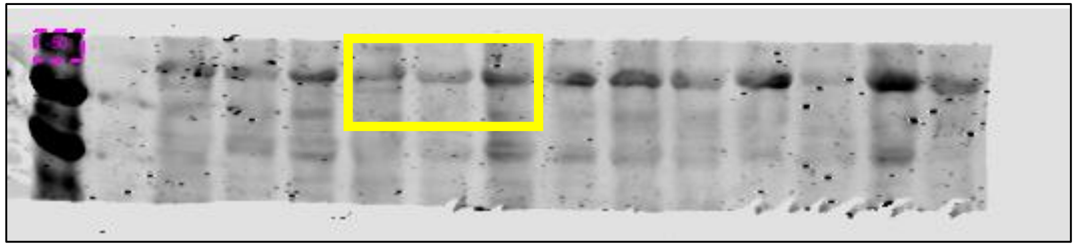

Phospho-c-met

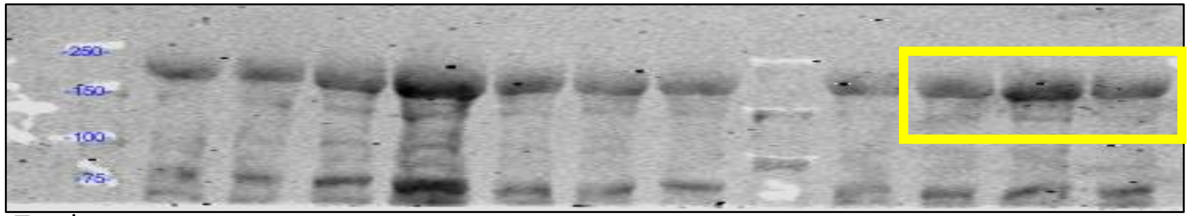

Total c-met

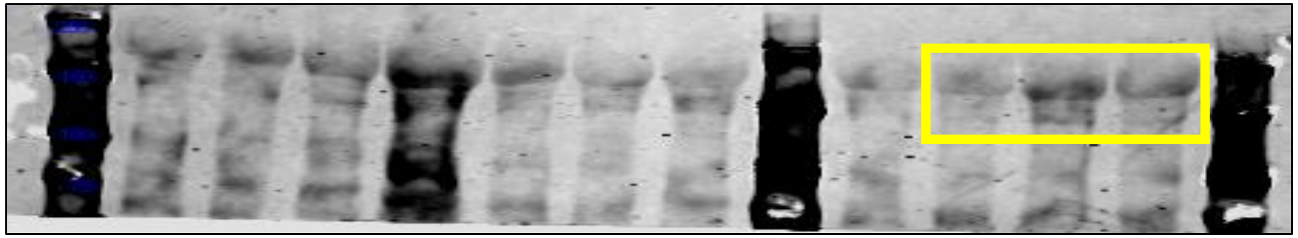

γ-Tubulin

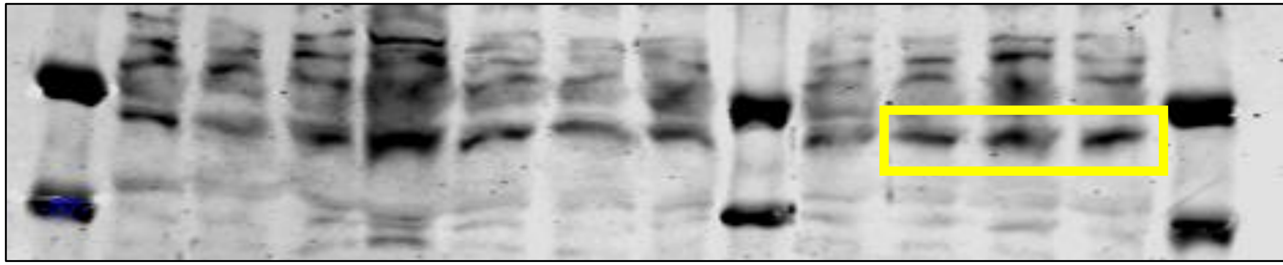

Supplement: Supplementary file 2 — Supplementary file2 (PDF 1026 KB) [file 210_2024_3436_MOESM2_ESM.pdf]
